# Single-band upconversion nanoprobes for multiplexed simultaneous *in situ* molecular mapping of cancer biomarkers

**DOI:** 10.1038/ncomms7938

**Published:** 2015-04-24

**Authors:** Lei Zhou, Rui Wang, Chi Yao, Xiaomin Li, Chengli Wang, Xiaoyan Zhang, Congjian Xu, Aijun Zeng, Dongyuan Zhao, Fan Zhang

**Affiliations:** 1 Department of Chemistry, iChEm (Collaborative Innovation Center of Chemistry for Energy Materials), State Key Laboratory of Molecular Engineering of Polymers, Fudan University, Shanghai 200433, China; 2 Obstetrics and Gynecology Hospital of Fudan University, Shanghai 200011, China; 3 Shanghai Institute of Optics and Fine Mechanics, Chinese Academy of Sciences, Shanghai 201800, China

## Abstract

The identification of potential diagnostic markers and target molecules among the plethora of tumour oncoproteins for cancer diagnosis requires facile technology that is capable of quantitatively analysing multiple biomarkers in tumour cells and tissues. Diagnostic and prognostic classifications of human tumours are currently based on the western blotting and single-colour immunohistochemical methods that are not suitable for multiplexed detection. Herein, we report a general and novel method to prepare single-band upconversion nanoparticles with different colours. The expression levels of three biomarkers in breast cancer cells were determined using single-band upconversion nanoparticles, western blotting and immunohistochemical technologies with excellent correlation. Significantly, the application of antibody-conjugated single-band upconversion nanoparticle molecular profiling technology can achieve the multiplexed simultaneous *in situ* biodetection of biomarkers in breast cancer cells and tissue specimens and produce more accurate results for the simultaneous quantification of proteins present at low levels compared with classical immunohistochemical technology.

## Abstract (web summary)

The excitation–emission profiles of upconversion nanoparticles (UCNPs) make them attractive biological probes. Here the authors present a lanthanide UCNP for the *in situ* multiplexed detection of cancer biomarkers, with different single-colour upconversion emissions.

## Graphical abstract

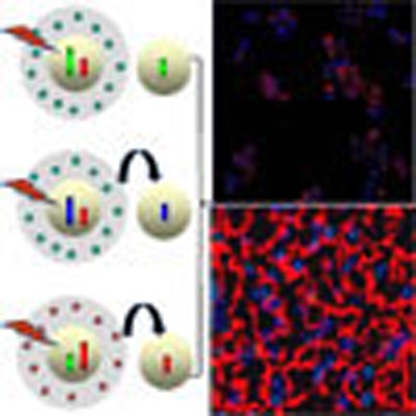

Multiplexed, sensitive and specific molecular detection is highly desirable for gene and protein profiling, drug screening, clinical diagnostics and environmental analysis^1^,^2^,^3^. For cancer diagnosis, the identification of potential diagnostic biomarkers and target molecules among the plethora of tumour oncoproteins requires facile technology capable of quantitatively analysing multiple biomarkers in tumour cells and tissues^4^,^5^,^6^,^7^,^8^. Diagnostic and prognostic classifications of human tumours are currently based on immunoenzyme-based immunohistochemical (IHC) methods with single-wavelength detection^9^, which are somewhat outdated and focus on approved antibodies for the IHC detection of cancerous markers. Furthermore, IHC remains semi-quantitative and subjective, resulting in considerable inter-observer and intra-observer variations in results^10^. Moreover, a major difficulty in molecular profiling is that most tumours (particularly breast and prostate) are highly heterogeneous and contain a mixture of benign cells, malignant cells, fibroblasts, stromal cells, vascular cells and infiltrating inflammatory cells (such as macrophages and lymphocytes)^11^. Current technologies such as reverse transcription–PCR, gene chips, protein chips, two-dimensional gel electrophoresis and mass spectrometry (for example, matrix-assisted laser desorption/ionization mass spectrometry (MALDI-MS), electrospray mass spectrometry (ES-MS) and surface-enhanced laser desorption/ionization mass spectroscopy (SELDI-MS)) are not suitable for analysing this type of heterogeneous sample^12^,^13^,^14^,^15^. A further limitation of these technologies is that they require destructive preparation of cells or tissue specimens as a homogeneous solution, leading to the loss of valuable three-dimensional cellular and tissue morphological information associated with the original tumour.

Compared with adsorption-based IHC methods, fluorescence imaging with optical microscopy provides a number of advantages, such as higher detection sensitivity, wider signal dynamic ranges and more linear relationships for biomarker quantification and prediction of therapeutic response. Indeed, recent advances have demonstrated that the fluorescence-based analysis of protein biomarkers with both fluorescent dye^16^ and quantum dots (QDs)^17^,^18^ is strongly correlated with clinical outcome. However, the use of organic dyes for multicolour fluorescence measurement is often limited by photobleaching and the need for multiple light sources to excite different fluorophores. QDs offer significant advantages over conventional fluorescent dyes because they are more photostable and can be excited in the same spectral range. However, optical interference of QDs with the autofluorescence background of biological tissue with the same excitations in the blue or ultraviolet region remains a major problem. To minimize this spectral interference, QD-stained specimens must be illuminated over an extended period of time to reduce the autofluorescence background before recording images and spectra (typically ranging from seconds to minutes depending on the intensity of the light source)^19^. Furthermore, because of overlap in the emission spectra of QDs and organic dyes, multiplexed simultaneous *in situ* detection requires image-processing techniques, such as spectral deconvolution^19^, which limit the screening rate and sensitivity. Thus, the development of appropriate multiplexed molecular profiling technologies remains a challenging task that is crucial for the development of cancer diagnosis technology, biosystem spatiotemporal analysis and the medical sciences.

An attractive alternative approach to this problem involves the use of lanthanide UCNPs, which can be excited in the tissue-transparent infrared region instead of at ultraviolet or visible frequency to produce photoluminescent emissions in the visible range (anti-Stokes process)^20^,^21^,^22^,^23^,^24^,^25^,^26^,^27^,^28^,^29^,^30^,^31^,^32^,^33^,^34^. Triply ionized lanthanide ions in hosts typically have emission line widths of 10–20 nm (full width at half maximum) in the visible portion of the spectrum, which is approximately half the line width observed for QDs (25–40 nm) and much narrower than the line width observed for organic dyes (30–50 nm)^17^,^18^,^32^. This feature increases the number of distinguishable emission bands within a specific spectral bandwidth, enabling a large number of multiplexed detections.

For *in situ* multiplexed molecular mapping, accurate molecular profiling can only be achieved with single-band emission nanoprobes by excluding crosstalk between different labelling signals. However, each lanthanide ion has a unique set of energy levels and generally exhibits a set of sharp emission peaks with distinguishable spectroscopic fingerprints^32^,^35^. Although some groups have reported that single-band upconversion emission can be realized by co-doping transition metal ions, only red (650–670 nm)^36^ and near infrared (NIR; ∼800 nm)^37^ single-band upconversion emissions have been realized. A general method to achieve more single-band upconversion emissions remains a major challenge for applications such as multiplexed intracellular detection and IHC staining without the constraints associated with conventional multi-peak UCNPs.

In this work, we report a general and simple method of achieving single-band upconversion emission with different colours by coating the upconversion nanocrystals with a screen layer containing an organic dye with a high molar absorption coefficient as nanofilters to remove the unwanted emission bands. As a result of the efficient reabsorption of the organic dye, remarkably pure single-band upconversion emissions can be generated in the blue, green and red regions. We use single-band UCNPs (sb-UCNPs) for the quantitative and simultaneous *in situ* profiling of multiple biomarkers in intact breast cancer cells and tissues. The biomarker expression levels in breast cancer cell specimens determined using sb-UCNPs, standard western blotting (WB) and IHC technology exhibit excellent correlation among these three methods; however, the WB and IHC methods can probe only one biomarker at a time. Significantly, the application of antibody-conjugated sb-UCNP molecular profiling technology can achieve the multiplexed simultaneous *in situ* biodetection of biomarkers in breast cancer cells and tissue specimens and produces more accurate results for the simultaneous quantification of proteins present at low levels compared with IHC.

## Results

### Fabrication of sb-UCNPs

**Figure 1:**
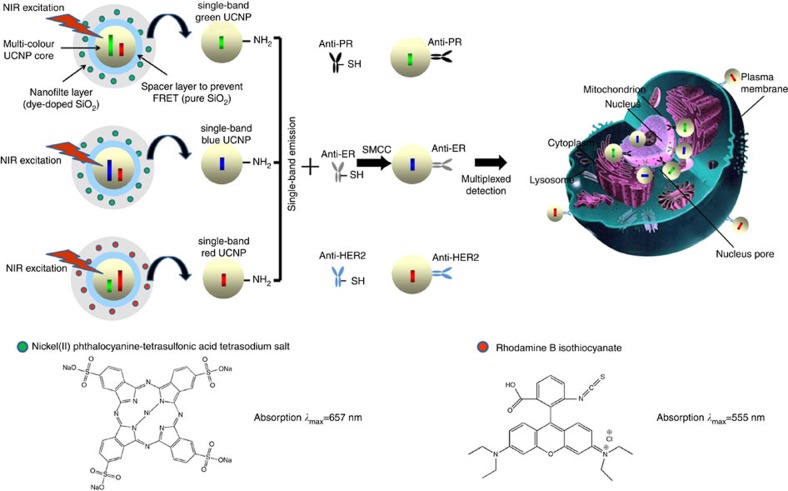
Schematic diagram of sb-UCNP fabrication for multiplexed detection. Surface amino modifications of the multi-layer structure of green, blue and red sb-UCNPs and conjugates with antibodies to the breast cancer biomarkers PR, ER and HER2, respectively, for multiplexed *in situ* molecular mapping of breast cancer biomarkers.

**Figure 2:**
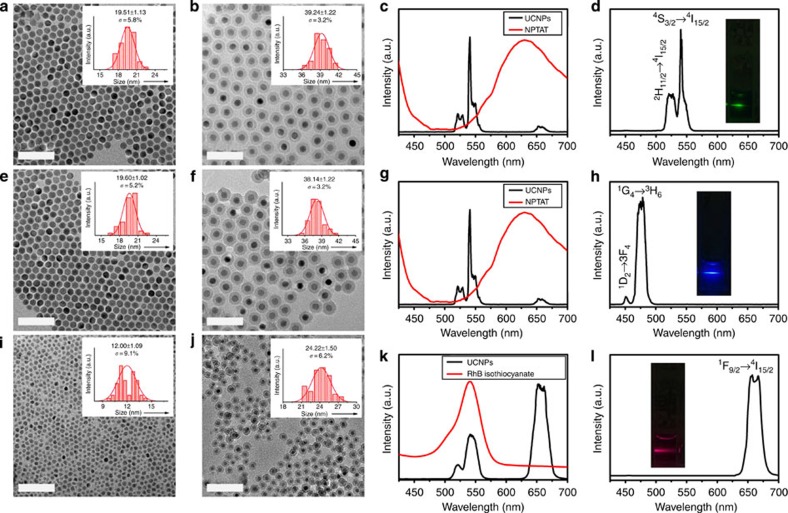
Fabrication and properties of single-band upconversion nanoprobes. TEM images and size distributions of (**a**) β-NaGdF_4_:20% Yb, 2% Er@NaGdF_4_ nanocrystals, (**b**) green emission sb-UCNPs (β**-NaGdF_4_:20% Yb, 2% Er@NaGdF_4_@SiO_2_@NPTAT-doped SiO_2_), (**e**) β-NaGdF_4_:20% Yb, 0.2% Tm@NaGdF_4_ nanocrystals, (**f**) blue emission sb-UCNPs (β**-NaGdF_4_:20% Yb, 0.2% Tm@NaGdF_4_@SiO_2_@NPTAT-doped SiO_2_), (**i**) α**-NaYbF_4_:10% Er@NaYF_4_ nanocrystals and (**j**) red emission sb-UCNPs (NaYbF_4_:10%Er@NaYF_4_@SiO_2_@rhodamine B isothiocyanate-doped SiO_2_). Upconversion photoluminescence spectra of the green, blue and red sb-UCNPs (**d**,**h**,**l**) and original upconversion nanocrystals measured in water and cyclohexane, respectively (**c**,**g**,**k**) (insets: corresponding photoluminescent photos of the colloidal solutions under continuous wavelength 980-nm laser excitation). Scale bars, 100μm.

The upconversion nanocrystals were prepared by the successive layer-by-layer strategy developed previously by our group (Supplementary Table 1)^38^. To obtain green emission sb-UCNPs, β-NaGdF_4_:20% Yb, 2% Er@NaGdF_4_ nanocrystals emitting both in the green (540 nm) and red (650 nm) were first prepared (Figs 1 and 2a,c). Then, the nanocrystals were coated with a pure silica spacer layer and amino-reactive organic dyes doped silica layer to form a selective nanofilter around the nanocrystals sequentially using a microemulsion method (Fig. 1; Fig. 2b). The organic dyes were selected based on the criteria of overlapping absorption spectra with only one of the dual-emission bands of the nanocrystals with a high molar absorption coefficient on the scale of 10^5^ M^−1^ cm^−1^. The pure silica spacer layer was used to prevent fluorescence resonance energy transfer (FRET) between the filtered upconversion emission band and the fluorescent dye-doped screen layer. To obtain green single-band emission, nickel (II) phthalocyanine-tetrasulfonic acid tetrasodium salt (NPTAT) organic dyes with a maximum absorption wavelength (*λ*_max_) of 657 nm were added with tetraethyl orthosilicate (TEOS) to form NPTAT-doped silica nanofilters on the β-NaGdF_4_:20% Yb, 2% Er@NaGdF_4_@SiO_2_ nanoparticles to filter the red emission band efficiently. With the β-NaGdF_4_:20% Yb, 2% Er@NaGdF_4_@SiO_2_@NPTAT-doped SiO_2_ nanostructure, only the narrow green emission centred at 540 nm was observed (Fig. 2d; Supplementary Fig. 1), in stark contrast to the dual-upconversion emission bands of β-NaGdF_4_:20% Yb, 2% Er@NaGdF_4_ (Fig. 2c). To obtain the blue and red emission sb-UCNPs, β-NaGdF_4_:20% Yb, 0.2% Tm@NaGdF_4_ and α-NaYbF_4_:10% Er@NaYF_4_ nanocrystals with strong blue to red (Fig. 2e,g) and red to green (Fig. 2i,k) upconversion emission ratios were first prepared. After coating the pure SiO_2_ layers, nanofilters doped with NPTAT and rhodamine B isothiocyanate (Fig. 2f,j) were used to filter the red and green emissions to obtain the final blue and red sb-UCNPs, respectively (Fig. 2h,l; Supplementary Figs 2–4). This general approach permits not only the removal of minor emission peaks away from the main peaks using appropriate nanofilters but also the alternative removal of the main peaks to leave the minor peaks for single-band upconversion emission. For example, green emission sb-UCNPs can be obtained by coating the α-NaYbF_4_:10% Er@NaYF_4_ nanocrystals with NPTAT-doped nanofilters (Supplementary Fig. 5).

In accordance with the Lambert–Beer law, the filtered intensity can be increased by increasing the dye concentration in the screen layer at a given wavelength^39^. Therefore, the intensity of a specific upconversion emission band can be reduced gradually by increasing the dye concentrations. For example, to obtain blue, green and red emission sb-UCNPs under 980-nm laser excitation (10 W cm^−2^), the molar ratio of doped dyes to lanthanide luminescent centres (Tm or Er) should be adjusted to 9:5, 1:5 and 11:100, respectively (Supplementary Table 2; Supplementary Figs 6–9). In addition, we observed that dye-doped nanofilters can be coated directly on the upconversion nanocrystals to achieve single-band upconversion emission when non-fluorescence dyes are used (Supplementary Fig. 10). However, FRET between the upconversion emission and the dyes with overlapping absorptions cannot be avoided if fluorescent dye-doped nanofilters are used. To obtain sb-UCNPs with high chromatic purities, a silica spacer layer must be inserted before coating the fluorescent dye-doped nanofilter layer (Supplementary Fig. 11a,b). Because the FRET effect is dependent on the distance between the donor and acceptor^40^, the thickness of the silica spacer layer should be adjusted accordingly. In this work, we determined that the FRET effect can be prevented completely by a 5-nm silica spacer layer for sb-UCNPs with different colours.

### Optical properties of sb-UCNPs

**Figure 3:**
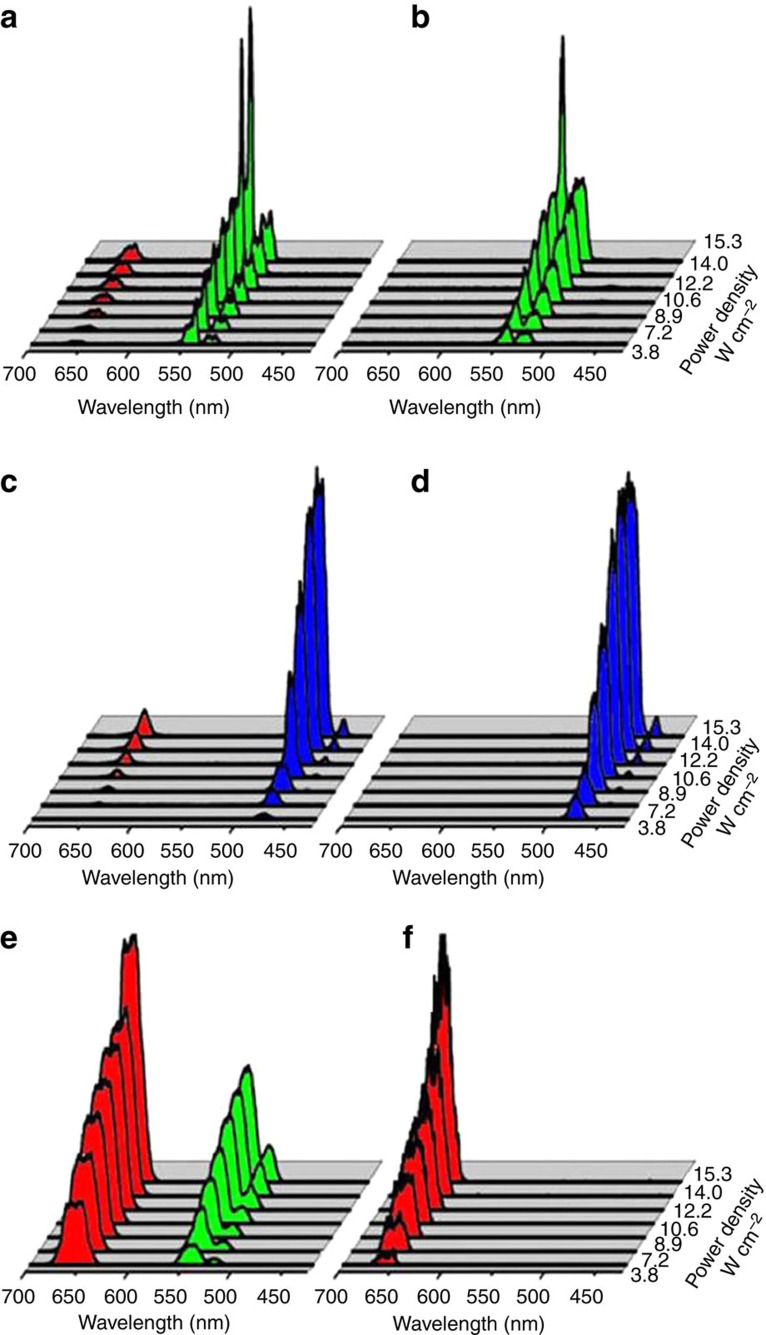
Optical properties of single-band upconversion nanoprobes. Pump-power-dependent upconversion photoluminescent emission spectra of the green, blue and red sb-UCNPs (**b**,**d**,**f**) and original upconversion nanocrystals (**a**,**c**,**e**) measured in water and cyclohexane, respectively. Note that the single-band features of the sb-UCNPs with the nanofilters are independent of the excitation laser power.

In the upconversion photoluminescence processes, the visible output intensity is proportional to the *n*th power of the NIR excitation intensity, that is, *I*_up_∝*(I*_exc_)^*n*^, where *I*_up_ and *I*_exc_ represent the upconversion intensity and the NIR excitation intensity, respectively, and *n* is the number of photons required to produce an upconversion photon^41^. Figure 3 shows the power dependence of the upconversion intensities of the sb-UCNPs and original upconversion nanocrystals. By increasing the pump power density up to 15.3 W cm^−2^, the green, blue and red single-band emission intensities were increased accordingly, but the single-band feature of the sb-UCNPs was maintained with increasing excitation power density (Fig. 3b,d,f). Significantly, the single-band feature of the sb-UCNPs was even maintained at the saturation point of upconversion emission intensity under high excitation power density (82 W cm^−2^; Supplementary Fig. 12). By contrast, before nanofilter coating, the original β-NaGdF_4_:20% Yb, 2% Er@NaGdF_4_, β-NaGdF_4_:20% Yb, 0.2% Tm@NaGdF_4_ and α-NaYbF_4_:10% Er@NaYF_4_ nanocrystals exhibited multipeak emissions with relative intensity ratios closely associated with the pump power density (Fig. 3a,c,e).

**Figure 4:**
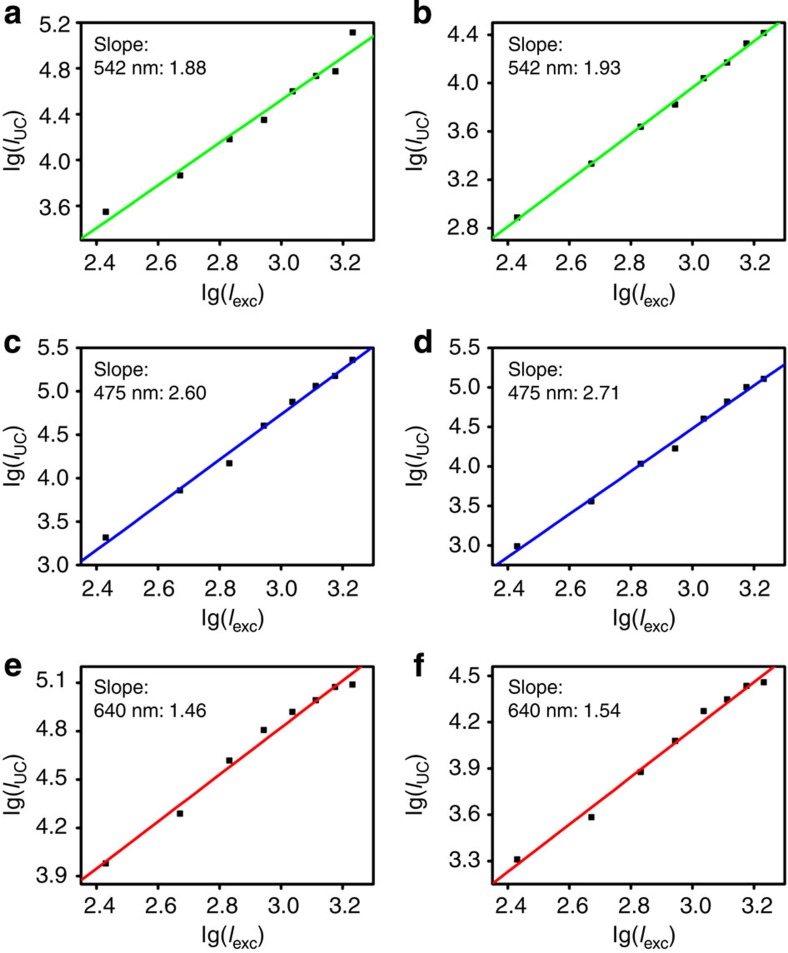
Power-dependent measurement of single-band upconversion nanoprobes. Power density dependence of upconverted emission of original upconversion nanocrystals (**a**,**c**,**e**) and the three single-band upconversion nanoprobes (**b**,**d**,**f**) at 475, 542 and 640 nm.

The slopes of the log–log plots of emission intensity versus NIR excitation power for the sb-UCNPs were 1.93 (green, ^2^H_11/2_, ^4^S_3/2_-^4^I_15/2_ transition), 2.71 (blue, ^1^G_4_-^3^H_6_ transition) and 1.54 (red, ^4^F_9/2_-^4^I_15/2_ transition; Fig. 4b,d,f), revealing little variation compared with the original upconversion nanocrystals (1.88 (green), 2.60 (blue) and 1.46 (red); Fig. 4a,c,e). This result indicates that the nanofilter coating did not affect the electronic transition states of lanthanide and that multi-photon features were maintained for the sb-UCNPs. Furthermore, the lifetimes of the three single-band emissions were 1066±17 μs (blue), 89±1 μs (green) and 59±1 μs (red), revealing little variation compared with the pure silica-coated UCNPs (1045±14 μs (blue), 88±3 μs (green) and 55±3 μs (red); Supplementary Fig. 13), which indicates that the phosphorescence properties of the sb-UCNPs are maintained after coating with the nanofilters.

**Figure 5:**
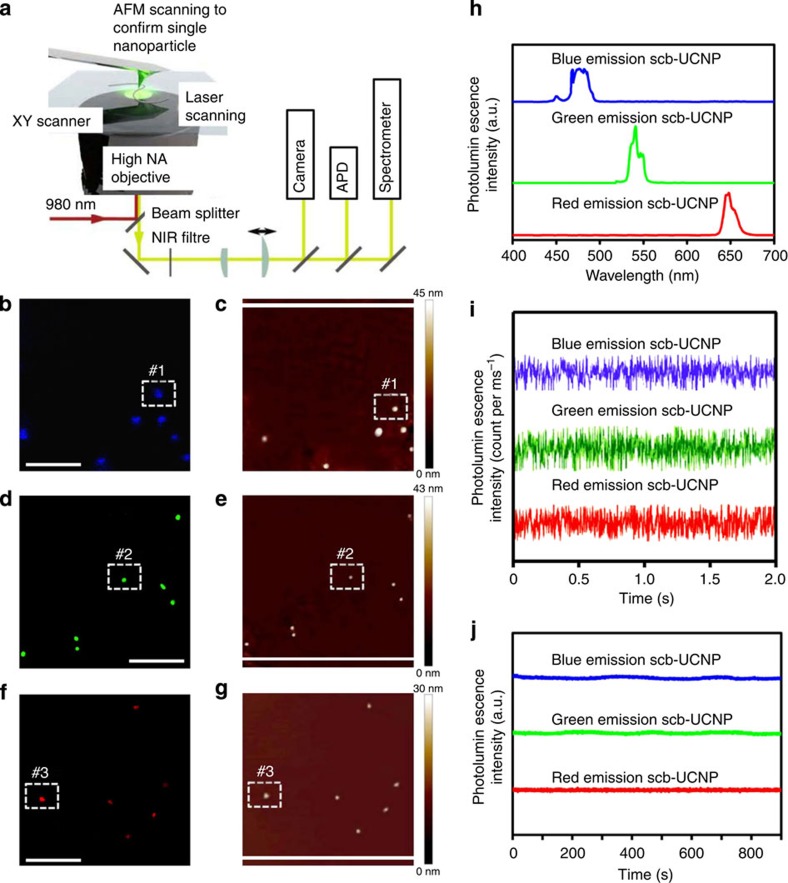
Single-particle optical property measurements of sb-UCNPs. (**a**) Experimental setup. On top of an inverted microscope, an AFM allows direct size determination combined with optical characterization. Upconversion photoluminescence images (**b**,**d**,**f**) and AFM images (**c**,**e**,**g**) of blue (**b**,**c**), green (**d**,**e**) and red (**f**,**g**) emission sb-UCNPs dispersed on a poly(L-lysine)-coated coverslip. Both photoluminescence and AFM images were obtained for the same sample region, as indicated by the 1:1 correspondence of the particle locations. (**h**) Typical spectra of single sb-UCNPs #1, #2# and #3. Emission bands corresponding to the transitions ^1^G_4_→^3^H_6_ (∼480 nm), ^4^S_3/2_→^4^I_15/2_ (∼550 nm), and ^4^F_9/2_→^4^I_15/2_ (∼650 nm) can be observed. (**i**) The photoluminescence time traces for single nanoparticles #1, #2 and #3 acquired with 10-ms time bins. (**j**) Long-lifetime photoluminescence intensity traces for single nanoparticles #1, #2 and #3 acquired with 500-ms time bins. All optical data shown were obtained with continuous wavelength 980-nm laser excitation. Scale bar, 5 μm.

To determine the photostability of the sb-UCNPs, dilute samples of sb-UCNPs with different emission colours were dispersed on the coverslips and imaged by atomic force microscopy (AFM)-coupled optical microscopy with excitation with a tightly focused 980-nm continuous wave (CW) laser (Fig. 5a)^31^,^42^. Upconverted photoluminescent images of the samples revealed homogeneous and randomly distributed diffraction-limited spots corresponding to either single particles or aggregates (Fig. 5b,d,f). In addition, as confirmed by the 1:1 correspondence of the optical and AFM images (Fig. 5c,e,g), particles #1 to #3 were unambiguously assigned as single nanoparticles with different colours. The photoluminescence emissions (Fig. 5h) and time traces (Fig. 5i,j) for particle #1 to #3 were studied under continuous illumination with a 980-nm CW laser. Notably, no photoblinking was detected when the bin time for each data point in emission intensity was reduced to 10 ms (Fig. 5i). Furthermore, the sb-UCNPs investigated here did not exhibit photobleaching or photodamage after 15 min of continuous laser irradiation with a 980-nm CW laser (Fig. 5j).

The photostability of the NPTAT and rhodamine B isothiocyanate dyes doped in nanofilters was quantitatively tested in both sb-UCNPs and NaGdF_4_ or NaYbF_4_ nanoparticles devoid of emitting lanthanides (Supplementary Fig. 14). The optical absorption peaks for both organic dyes were maintained under 980-nm laser excitation for 30 min. Because the upconversion quantum yield of UCNPs is low (<1%)^20^,^40^, it is not adequate to evaluate the photostability of the organic dyes doped with nanofilters under upconversion visible emission. Therefore, a xenon lamp (500 W) with a prism spectroscope was used to generate higher-power visible irradiation overlapping with the absorption wavelength of the NPTAT and rhodamine B isothiocyanate dyes (Supplementary Fig. 14). The optical absorption peaks for both organic dyes were maintained under xenon lamp excitation for 30 min. The above results indicate that the homogeneous doping of organic dyes in the silica layer did not alter the photostability of the organic dyes under the 980-nm NIR laser or visible light irradiation.

### Multiplexed detection for cancer cells and tissue specimens

**Figure 6:**
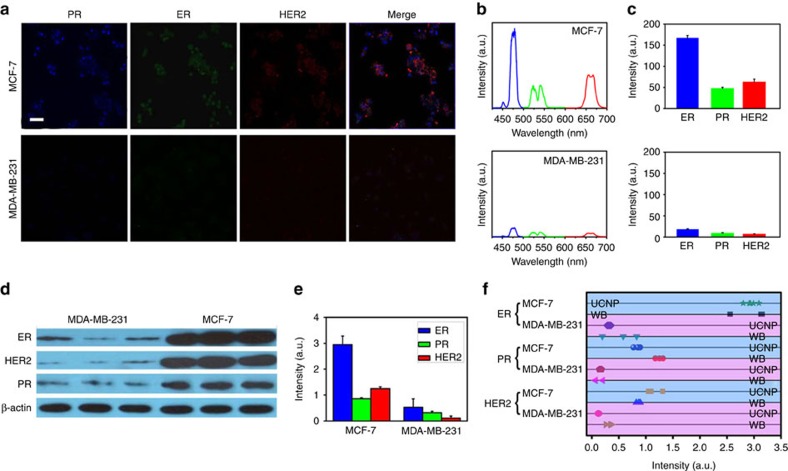
Multiplexed detection in breast cancer cell lines using sb-UCNPs. (**a**) Expression of PR, ER and HER2 signals in MCF-7 and MDA-MB-231 breast cancer cells detected by fluorescence microscopy (pseudo-colour). (**b**) Representative spectra of the protein biomarkers obtained by single-cell spectroscopy measurements. (**c**) Average expression levels of ER, PR and HER2 determined by spectral analysis of 100 single cells. Data points represent mean+s.d. (*n*=100). (**d**,**e**) ER, PR, and HER2 expression in MCF-7 and MDA-MB-231 cells as measured by WB. Data points represent mean+s.d. (*n*=3). (**f**) Comparative statistical analysis of sb-UCNPs-Abs bioconjugate profiling data and WB results. Scale bar, 10 μm.

To demonstrate the feasibility of the multiplexed labelling, multicolour sb-UCNPs emitting at 480, 540 and 650 nm were directly conjugated with primary antibodies (Abs) against the three most important breast cancer biomarkers (oestrogen receptors (ERs), progesterone receptors (PRs) and human epithelial growth fator receptor-2 (HER2)), which are routinely analysed in surgical pathology laboratories and on which therapeutic decisions are based (Fig. 1). MCF-7 and MDA-MB-231 breast cancer cells, which have different expression levels of the three protein markers, were stained with sb-UCNP-Abs bioconjugates (sb-UCNPs-Anti-PR (green), sb-UCNPs-Anti-ER (blue) and sb-UCNPs-Anti-HER2 (red)). Using multispectral confocal microscopy, spectrally separated upconversion fluorescence was clearly visible in both cell lines (Fig. 6a; Supplementary Fig. 15; Supplementary Fig. 16). The remarkable photostability of the sb-UCNPs with low biological background fluorescence under 980-nm excitation allows extended exposure to excitation lights and consequently allows the reconstruction of multicolour projection. As shown in Fig. 6a, HER2 was located on the cell membrane, and both ER and PR were detected predominantly in the cell nuclei. These results demonstrate that sb-UCNP-Abs bioconjugates can be used to detect proteins regardless of their cellular location. To further explore whether fluorescent signals from labelled proteins can be simultaneously quantified, we performed single-cell spectroscopy using a wavelength-resolved spectrometer coupled with a confocal microscope. Individual spectra of each labelled protein with peaks in the region of the sb-UCNP emission maxima (480, 540 and 650 nm) were observed, representing the photoluminescent emission of the sb-UCNP-Abs (Fig. 6b). Due to the narrow upconversion emission bands, there was no spectral overlap for the multiplexed simultaneous detection of the three protein markers with sb-UCNPs, which greatly overcomes the limitations of QDs and organic dyes with clear multicolour emission spectral overlap^43^,^44^.

Without adjusting the photoluminescence intensities of individual colours, the relative photoluminescence intensities of a biomarker across different cell lines are more meaningful than the comparison of the photoluminescence intensities of different biomarkers in one type of cells. This distinction is the result of the differential optical and structural properties of sb-UCNPs of different colours. The use of different photoluminescence detectors in different labs can also lead to discrepancies in experiment results. We observed that the 480- and 540-nm sb-UCNPs were 1.1-fold and 1.5-fold, respectively, brighter than the 650-nm sb-UCNPs at the same concentration (Supplementary Fig. 17). When the individual colours were resolved using a previously reported method^19^,^45^ and normalized against this ‘brightness index', the peaks at 650 and 480 nm were increased 1.5-fold and 1.3-fold with respect to their original values, respectively, whereas the peak at 540 nm was unchanged. The results obtained by quantitative spectrometry (Fig. 6b) correlated well with the biomarker expression patterns obtained using the traditional WB technique (Fig. 6d,e). For statistical significance, the photoluminescence spectra of 100 cells in each cell line were measured, and the mean levels of HER2, ER and PR expression were evaluated (Fig. 6c). The protein expression results obtained using sb-UCNPs-Abs molecular profiling technology and WB were then compared after the absolute measurements of the triplet (HER2, ER and PR) were transformed to a relative scale in percent (Fig. 6f). Note that biomarker expression levels determined using sb-UCNP molecular profiling technology and standard WB for a given cell line tend to aggregate, revealing excellent correlation between the two methods.

**Figure 7:**
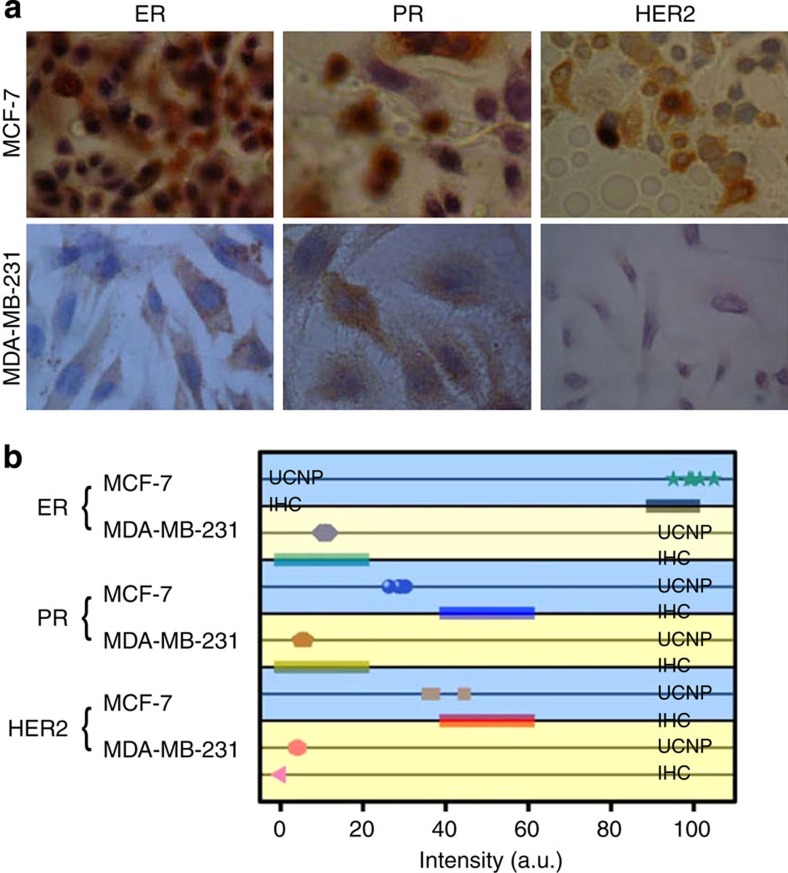
Comparison of multiplexed detection with sb-UCNPs and IHC. (**a**) Expression of ER, PR and HER2 in the breast cancer cell lines as estimated by IHC and (**b**) comparative statistical analysis of the sb-UCNP profiling data and IHC results.

Furthermore, because IHC is also one of the widely used methods for analysing cancer-related proteins, we compared the sb-UCNP molecular profiling technology with a traditional IHC method using the same set of breast cancer cell lines (Fig. 7a,b). The slides of cells were photographed after immunostaining in accordance with standard pathological protocols for ER, PR and HER2 (Fig. 7a; Supplementary Table 3). The IHC results were analysed by three independent observers and scored with a standard scale from 0 to 3+. Cells with IHC scores of 3+ had 100% relative expression of the proteins by sb-UCNP multiplexed detection (the highest expression level for each biomarker was set as unity). ER and PR expression in MDA-MB-231 cells were both classified as 1+ by IHC, which corresponded to 11 and 5% estimated by the new sb-UCNP molecular profiling technology (Fig. 7b; Supplementary Table 3), while the 4% HER2 expression detected by sb-UCNPs was classified with a 0 IHC score (Fig. 7b). In both cases, the expression of the proteins using sb-UCNPs correlated well with the IHC results. However, the application of conjugated sb-UCNPs and quantitative spectroscopy may be more accurate than IHC for quantifying proteins present at low levels.

The multiplexed detection results for cell lines indicate that the novel sb-UCNP-based molecular profiling technology is capable of imaging and quantifying multiple tumour biomarkers in intact tumour specimens. We therefore sought to apply this technology to detect PR, ER and HER2 biomarkers in FFPE human breast cancer specimens with Ab-conjugated sb-UCNPs. Furthermore, according to the American Society of Clinical Oncology/College of American Pathologists, the validated techniques to assess these biomarkers are IHC^46^. In light of this novel sb-UCNP-based molecular profiling technology, our aim was to perform a comparison study of PR, ER and HER2 concordance between IHC and sb-UCNP molecular profiling technology.

**Figure 8:**
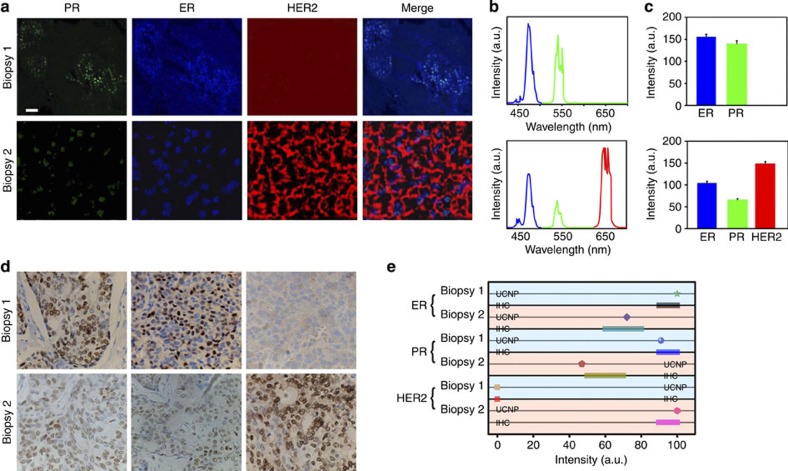
Multiplexed detection in breast cancer tissue specimens using sb-UCNPs. (**a**) Nuclear and cell membrane fluorescent signals were detected by microscopy (pseudo-colour), revealing the expression of ER, PR and HER2 biomarkers as quantified by wavelength-resolved spectroscopy. (**b**) Emission spectra resolved into individual channels and compensated for the differential brightness between different colours. (**c**) Average expression levels of ER, PR and HER2 calculated from the results of spectral analysis obtained by detecting 30 randomly selected spots in the tissue sections. Data points represent mean+s.d. (*n*=30). (**d**) Expression of ER, PR and HER2 estimated by IHC in the breast cancer tissue specimens and (**e**) comparative statistical analysis of the sb-UCNP profiling data and IHC results. Scale bar, 20μm.

In tumour biopsy 1, only PR and ER signals were observed by confocal microscopy (Fig. 8a), corresponding to the two upconversion photoluminescent peaks detected on the spectrogram (Fig. 8b). Bioimaging and spectroscopic detection of biopsy 2 revealed expression of PR, ER and HER2 (Fig. 8a,b). Quantification detection by analysing 30 randomly selected spots in the tissue sections with sb-UCNP molecular profiling technology revealed a definitive difference in HER2 expression between the two tumour specimens. Biopsy 1 did not express HER2, in contrast to the high expression of HER2 (147±10) in biopsy 2 (Fig. 8c; Supplementary Table 4). When compared with the IHC results using relative scales (Fig. 8d; Supplementary Table 4), the average values of biomarker expression measured using sb-UCNPs revealed excellent correlation for high-level protein expression (Fig. 8e; Supplementary Table 4). Similar to the cell line detection, the application of Ab-conjugated sb-UCNPs and quantitative spectroscopy may be more accurate at quantifying proteins than IHC in tumour specimens. For example, ER and PR expression in biopsy 2 were both classified as 2+ by IHC. However, more accurate detection was achieved for ER (72%) and PR (47%) expression using the new sb-UCNP molecular profiling technology.

## Discussion

If cancer could be reliably identified at the earliest possible stage of cellular transition, then novel therapeutics could be designed to arrest the process of malignant transformation. Aggressive behaviours or phenotypes may be understood and predicted by a defining set of biomarkers. By critically defining the interrelationships among these biomarkers, cancer could be diagnosed and prognosis predicted based on a patient's molecular profile, leading to personalized and predictive oncology. That is, a unique molecular profile could be used to predict the tumour's invasive and metastatic potential, its ability to survive and grow under androgen-deprived, hypoxic and metabolic stress conditions, and the potential of certain cancer cells to evade host immune surveillance. Current diagnostic and prognostic classifications of human tumours are mainly based on the IHC method with single-wavelength detection, which is not compatible with multiplexed molecular profiling^47^. Recent advances have demonstrated that the fluorescence-based analysis of protein biomarkers is strongly correlated with clinical outcome. Compared with conventional biological labels, such as organic dyes, QDs and regular lanthanide labels with ultraviolet excitation, UCNPs have many advantages, including virtually zero auto-fluorescence background, which improves the signal-to-noise ratio, large anti-Stokes shifts, which facilitate the separation of photoluminescence from the excitation wavelength, narrow emission bandwidths, which facilitate multiplexed imaging, and high resistance to photobleaching for compatibility with long-term repetitive imaging. In addition, UCNPs are non-blinking, exhibit reduced light scattering and permit deep tissue penetration because their excitation lies in the NIR region within the optical transparency window^48^.

In this report, we have developed a general and simple method to achieve single-band upconversion emissions with different colours and high chromatic purities by coating the upconversion nanocrystals with organic dye-doped nanofilters^48^. As a result of the efficient reabsorption of the organic dye with a high molar absorption coefficient, remarkably pure single-band upconversion emissions can be generated in the blue (480 nm), green (550 nm) and red (650 nm) regions. Moreover, lanthanide ions are spectroscopically rich species, and most of the rare earth (RE) ions can exhibit upconversion, including Pr, Nd, Sm, Eu, Tb, Dy, Ho, Er and Tm. By adjusting different combinations of doping ions and host materials^20^,^38^,^40^, the upconversion emission wavelength (from ultraviolet–visible to NIR) and relative intensity of emission peaks can be effectively controlled to modulate the emission colours of the lanthanide-doped nanocrystals. Therefore, this method can be extended to produce additional sb-UCNPs in combination with one or more appropriate filter dye types.

Significantly, in this work, we have demonstrated the use of sb-UCNPs for the multiplexed detection of three tumour biomarkers in both cultured human breast cancer cells and paraffin-embedded clinical tissue sections. The simultaneous quantification of ER, PR and HER2 receptor expression levels in the breast cancer cell specimens correlated closely with the results of the traditional WB method. Furthermore, the application of conjugated sb-UCNPs and quantitative spectroscopy may be more accurate than IHC for the simultaneous quantification of proteins present at low levels in cancer cells and tissue specimens. Thus, sb-UCNP-based technology may be well suited to the molecular profiling of tumour biomarkers *in vitro* and represent a clinical translational application of upconversion nanomaterials for cancer prognosis. The ability to detect multiple target proteins in small samples of cancer tissues could enable more effective therapeutic decisions when used in combination with regular IHC methods. The next step is to conduct large-scale clinical studies to establish protocols and practices for sb-UCNP-based molecular pathology.

Moreover, fluorescence colour codes used in a broad range of fields have been among the most important methods for multiplexing, as demonstrated in this work. Although much narrower emission peaks can be achieved with UCNPs, the crowded spectral domain still limits detection numbers. Creating additional distinguishable coding dimensions should enhance multiplexed detectabilities. Recently, Jin *et al*.^49^,^50^ demonstrated that tunable luminescent lifetimes in the microsecond region can be exploited to code individual upconversion nanocrystals (τ-dots) that are independent of both colour and intensity and extend the optical multiplexing capability by adding a temporal dimension to photoluminescent signals. In terms of luminescence intensity-based multiplexing, sb-UCNPs are superior, and thus combining single-band upconversion and the temporal dimension of luminescence signals is expected to further extend the optical multiplexing capability for next-generation applications in the life sciences.

## Methods

### Synthesis of lanthanide upconversion nanocrystals

The upconversion nanocrystals, including hexagonal phase β-NaGdF_4_:20% Yb, 0.2% Tm, β-NaGdF_4_:20% Yb, 0.2% Tm@NaGdF_4_, β-NaGdF_4_:20% Yb, 2% Er, β-NaGdF_4_:20% Yb, 2% Er@NaGdF_4_, cubic phase α-NaYbF_4_:10% Er and α-NaYbF_4_:10% Er@NaYF_4_ nanocrystals, were prepared by the successive layer-by-layer strategy (see Supplementary Methods).

### Synthesis of sb-UCNPs

The sb-UCNPs were synthesized by a reversed-phase microemulsion method. In a typical synthesis of single red-emission sb-UCNPs, a mixing solution of 0.5 ml of surfactant CO-520 (purchased from Sigma Aldrich), 16 ml of cyclohexane (purchased from Sinopharm Chemical Reagents Co. Ltd) and 1 ml of 0.1 Mmol α-NaYbF_4_:10% Er@NaYF_4_ solution in cyclohexane was vigorously stirred for 15 min. Next, 2.1 ml of CO-520 and 0.2 ml of NH_3_·_2_O (30 wt%, purchased from Beijing Chemical Reagents Co. Ltd) were added to the solution, and the container was sealed and sonicated for 15 min until a transparent reversed-phase microemulsion was formed. Then, 0.1 ml of TEOS (purchased from Sigma Aldrich) was added to the solution. After vigorous stirring for 2 h, 0.1 ml of (3-Aminopropyl) triethoxysilane (APTES) (purchased from Sigma Aldrich)-modified rhodamine B isothiocyanate (purchased from Sigma Aldrich) solution (5 mg ml^−1^) was added. After continuous stirring at room temperature at 750 r.p.m. for 2 days, the products were precipitated by adding ethanol. The collected precipitates were washed twice with ethanol and dispersed in ethanol for further use. The blue and green emission sb-UCNPs were synthesized in a similar manner using nickel (II) phthalocyanine-tetrasulfonic acid tetrasodium (5 mg ml^−1^, purchased from Sigma Aldrich) doping dyes.

### Wide-field luminescence and AFM imaging of single sb-UCNPs

To prepare an UCNP sample for single-particle measurements, a clean coverslip was first coated with poly(L-lysine; 0.1% w/v in H_2_O), and the sb-UCNP solution was then dispersed in it for measurement. We imaged the sb-UCNPs using an in-house wide-field fluorescence imaging setup based on an inverted microscope (X71, Olympus), an NIR excitation source (980-nm CW laser), and a highly sensitive ARC-SP 2360 spectrometer equipped with a Pro EMCCD camera (Pro EM: 1,600 × 200 B/l eXcelon EMCCD Camera System, Princeton Instruments, Inc.). The output of a 980-nm laser (SDL-980LM-500T, 500 mW, Shanghai Dream Lasers Technology) was spatially filtered using a 100-mm pinhole and collimated so that the beam diameter was ∼10 mm before entering the microscope. The beam was reflected by a short-pass optical filter (750SP from Chroma Corp) inside the microscope and directed to the microscope objective (numerical aperture 1.40, oil immersion, Olympus). After passing through the dichroic beam splitter, the emission was filtered by a short-pass emission filter (750SP from Chroma Corp) with a transmission range of 400–700 nm, which covers the blue, green and red emission bands of sb-UCNPs. The image of the sb-UCNP sample was further magnified using a set of achromatic lenses outside the microscope and finally detected by a Pro EMCCD camera. To determine whether the bright spots in such optical images corresponded to single sb-UCNPs or their aggregates, we imaged the same region by AFM (Catalyst, Bruker) and analysed the morphology of the particles.

### Multiplexed labelling of breast cancer cells

Breast cancer cell lines (MCF-7, MDA-MB-231) were obtained from American Type Culture Collection (ATCC, Manassas, VA, USA). For multiplexed staining of sb-UCNPs-Abs, cells (1 × 10^4^) were incubated in confocal dishes (35 × 10 mm) at 37 °C under 5% CO_2_ for 24 h. The cells were deparaffinized by incubation with formaldehyde (4%), followed by a 30-min incubation in a humidified chamber at 37 °C with 2% Triton X-100. After blocking with 5% bovine serum diluted in phosphate-buffered saline for 30 min, cells were stained with a mixture of conjugates targeting ER, PR and HER2 (40 nM each) for 1 h and washed five times in PBS.

### Quantitative spectrometry with confocal microscopy

Single-cell spectroscopy was accomplished using scanning mapping of a fluorescence confocal microscope. To measure biomarker expression in cultured cells, spatially separated individual cells were manually positioned in the ‘hot-spot' defined by the position and size of the pinhole. To determine biomarker expression in tissue specimens, sb-UCNP spectra from 30 randomly selected areas were measured. Data from the spectroscopic measurements were converted to ASCII format for further quantification and statistical analysis.

## Author contributions

F.Z. and L.Z. conceived the project and designed the experiments. L.Z., R.W., C.Y., X. L., X.Z., C.X., A.Z, D.Z. and F.Z. were primarily responsible for the data collection and analysis. F.Z. and L.Z. prepared figures and wrote the main manuscript text. All authors contributed to the discussions and manuscript preparation.

## Additional information

**How to cite this article:** Zhou, L. *et al*. Single-band upconversion nanoprobes for multiplexed simultaneous *in situ* molecular mapping of cancer biomarkers. *Nat. Commun*. 6:6938 doi: 10.1038/ncomms7938 (2015).

## Supplementary Material

Supplementary Information Supplementary Figures 1-17, Supplementary Tables 1-4, Supplementary Methods and Supplementary References
