# Supplementary material for: Single-band upconversion nanoprobes for multiplexed simultaneous in situ molecular mapping of cancer biomarkers
Source: Nat Commun. 2015 Apr 24;6:6938. doi: 10.1038/ncomms7938 (PMC4423208; doi:10.1038/ncomms7938)
Supplement: Supplementary Information — Supplementary Figures 1-17, Supplementary Tables 1-4, Supplementary Methods and Supplementary References [file ncomms7938-s1.pdf]

Supplementary Figures

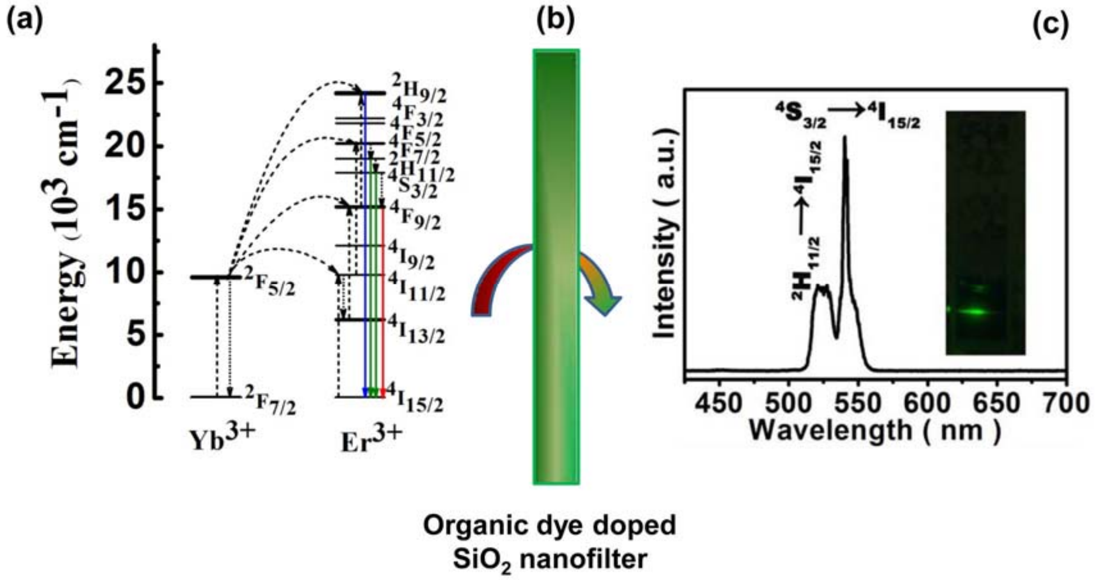

Supplementary Figure 1. The proposed luminescent mechanism of green sb-UCNPs. (**a**) Proposed energy transfer mechanism in the core/shell β-$NaGdF_4$: 20 % Yb, 2 % Er@$NaGdF_4$ UCNPs. (**b**) After coating the NPTAT-doped silica nanofilters on the β-$NaGdF_4$: 20 % Yb, 2 % Er@$NaGdF_4$@SiO_2 nanoparticles. (**c**) The red emission band can be filtered efficiently to realize the single-band green emission nanoparticles.

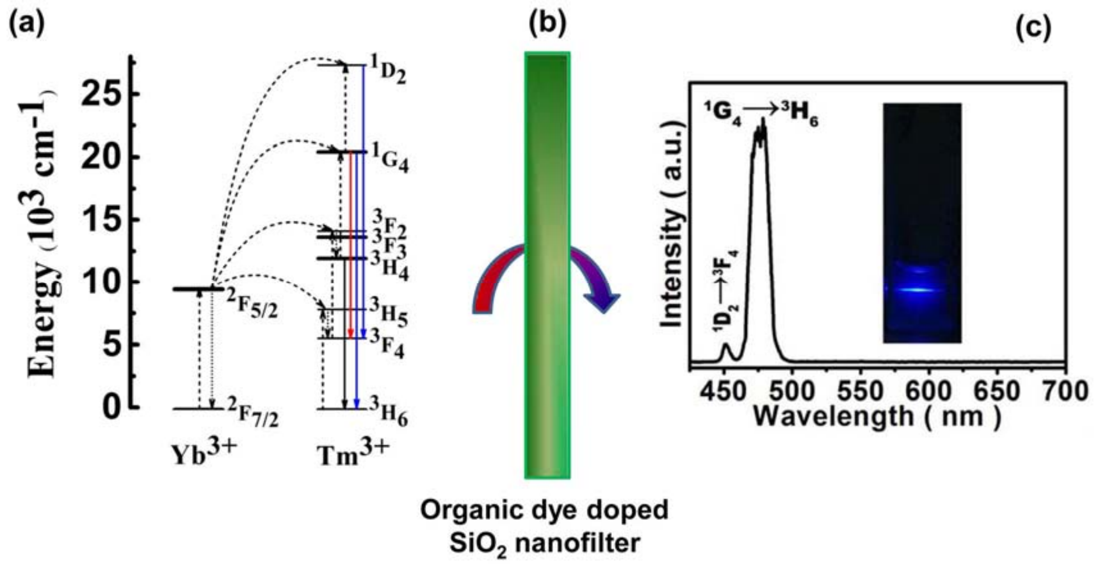

Supplementary Figure 2. The proposed luminescent mechanism of blue sb-UCNPs. (**a**) Proposed energy transfer mechanism in the core/shell *β*-NaGdF$_4$: 20 % Yb, 0.2 % Tm@NaGdF$_4$ nanoparticles. (**b**) After coating the NPTAT-doped silica nanofilters on the *β*-NaGdF$_4$: 20 % Yb, 0.2 % Tm@NaGdF$_4$@SiO$_2$ nanoparticles. (**c**) The red emission band can be filtered efficiently to realize the single-band blue emission nanoparticles.

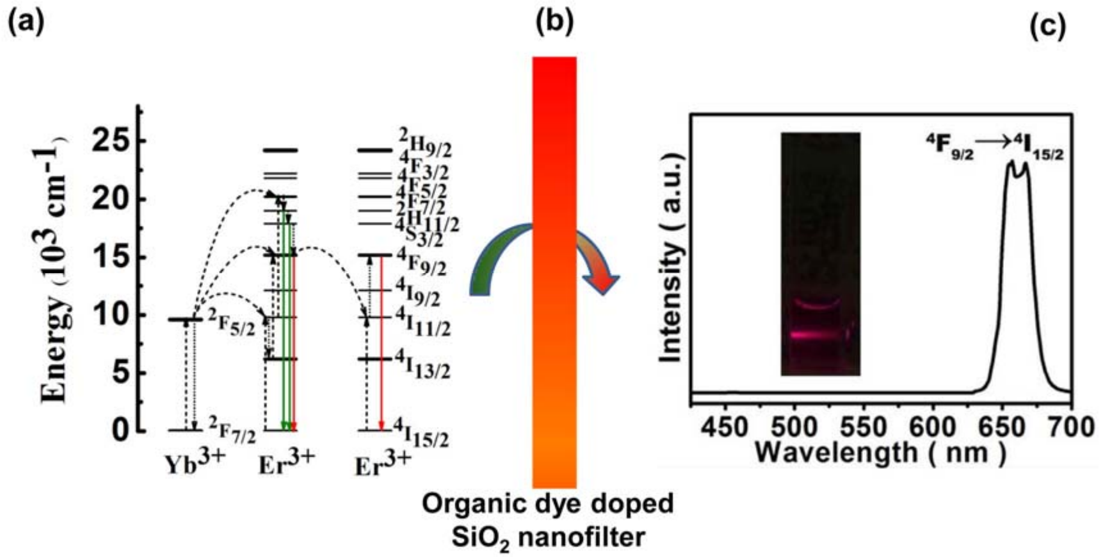

Supplementary Figure 3. The proposed luminescent mechanism of red sb-UCNPs. (**a**) Proposed energy transfer mechanism in the core/shell α-$NaYbF_4$: 10 % Er@$NaYF_4$ nanoparticles. (**b**) After coating the rhodamine B isothiocyanate-doped silica nanofilters on the α-$NaYbF_4$: 10 % Er@$NaYF_4$@SiO_2 nanoparticles. (**c**) The green emission band can be filtered efficiently to realize the single-band red emission nanoparticles.

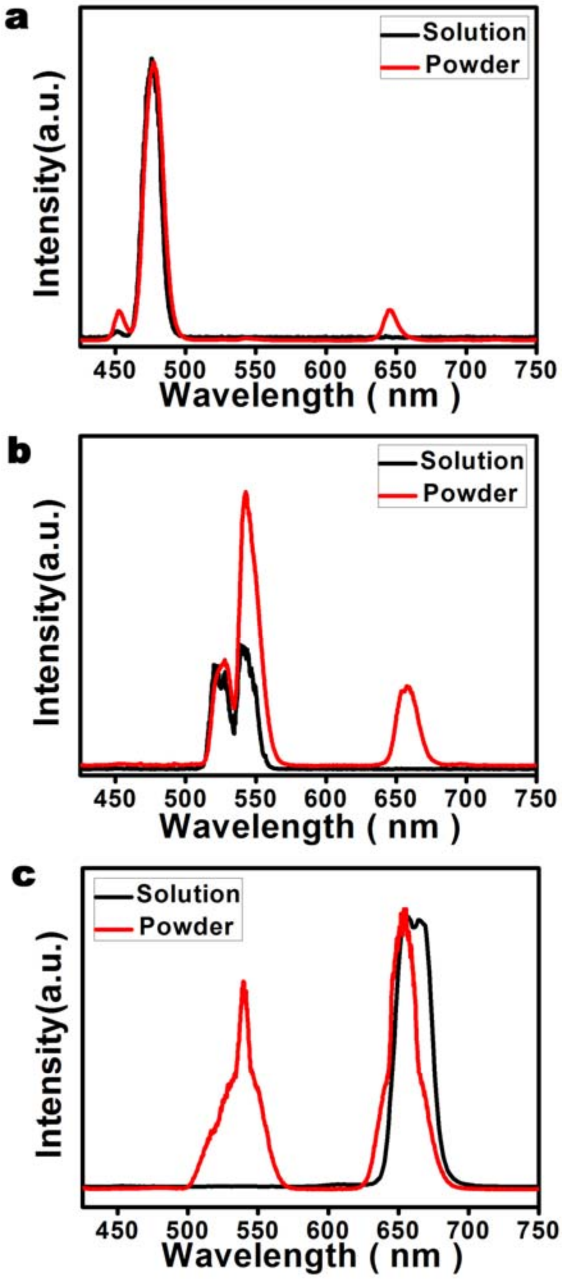

Supplementary Figure 4. Optical properties of materials at different states. Upconversion photoluminescence spectra of the blue (**a**), green (**b**) and red emission (**c**) sb-UCNPs measured with aqueous solution (deionized water) or powder samples, indicating that the single-band upconversion cannot be realized for the powder sample.

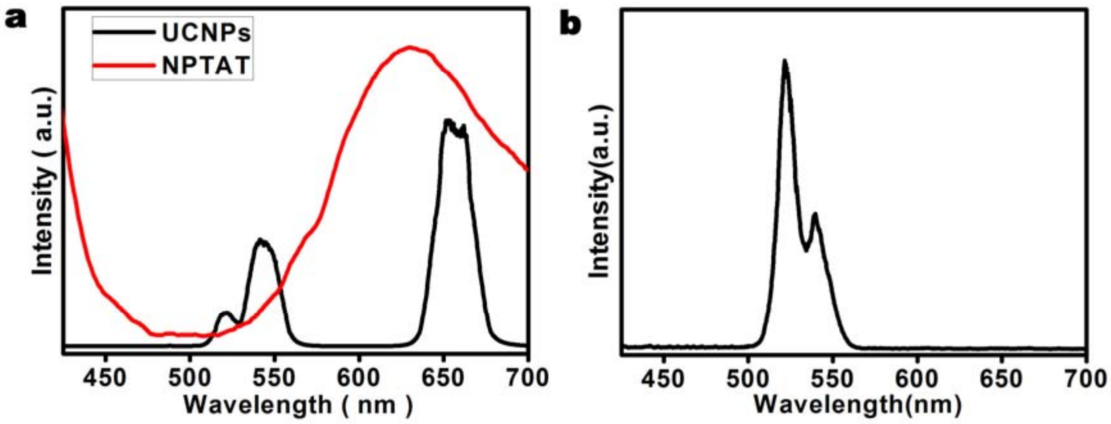

Supplementary Figure 5. Remove the main emission peaks of original UCNPs. (**a**) α-$NaYbF_4$: 10 % Er@$NaYF_4$ nanoparticles. After coating the NPTAT-doped silica nanofilters (**b**) on the α-$NaYbF_4$: 10 % Er@$NaGdF_4$@SiO_2 nanoparticles, the red emission band can be filtered efficiently to realize the green emission sb-UCNPs.

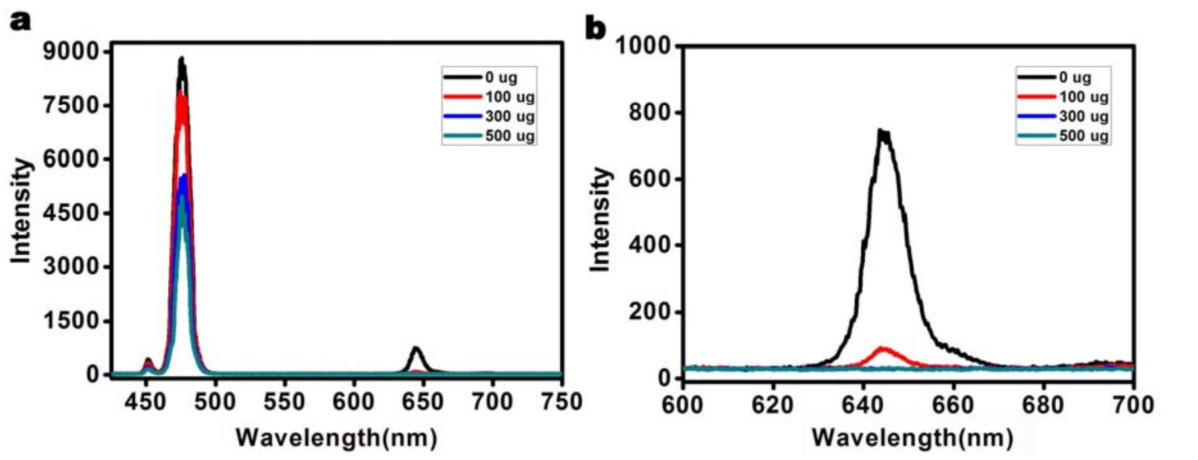

Supplementary Figure 6. Photoluminescence as a function of dye feeding amount. Upconversion photoluminescence spectra of β-$NaGdF_4$: 20 % Yb, 0.2 % Tm@SiO_2 with varied feeding amount of NPTAT. (**a**) β-$NaGdF_4$: 20 % Yb, 0.2 % Tm@SiO_2 nanoparticles with different feeding amount of NPTAT under 980 nm excitation (10 W/cm^2). (**b**) Enlargement figure of the 650 nm UC emission peaks with varied concentration of organic dyes. The numeral in the figure shows the feeding amount of NPTAT in the silica coating process; the doping amounts corresponding to the feeding amounts are shown in the **Supplementary Table 2.**

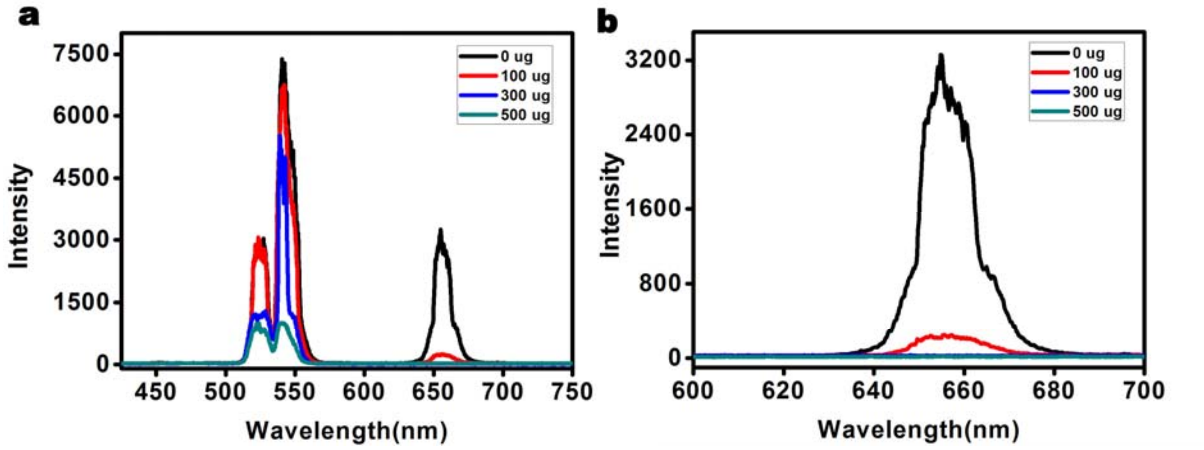

Supplementary Figure 7. Photoluminescence as a function of dye feeding amount. Upconversion photoluminescence spectra of *β*-$NaGdF_4$: 20 % Yb, 2 % Er@SiO_2 with varied feeding amount of NPTAT. (a) *β*-$NaGdF_4$: 20 % Yb, 2 % Er@ SiO_2 with different feeding amount of NPTAT dyes under 980 nm excitation (10 W/cm^2). (b) Enlargement figure of the 650 nm UC emission peaks with varied concentration of organic dyes. The numeral in the figure shows the feeding amount of NPTAT in the silica coating process, the doping amounts corresponding to the feeding amounts are shown in the **Supplementary Table 2.**

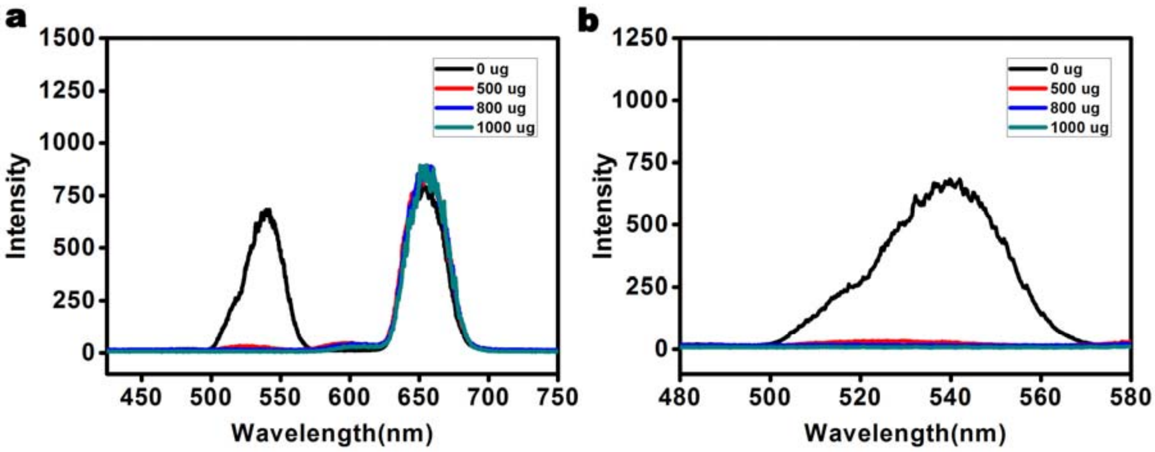

Supplementary Figure 8. Photoluminescence as a function of dye feeding amount. Upconversion photoluminescence spectra of α-$NaYbF_4$, 10 % Er@SiO_2 with varied feeding amount of rhodamine B isothiocyanate. (**a**) *α*-$NaYbF_4$, 10 % Er@SiO_2 nanoparticles with different feeding amount of rhodamine B isothiocyanate under 980 nm excitation (10 W/cm^2). (**b**) Enlargement figure of the 540 nm UC emission peaks with varied concentration of rhodamine B isothiocyanate. The numeral in the figure shows the feeding amount of APTES modified rhodamine B isothiocyanate in the silica coating process, the doping amounts corresponding to the feeding amounts are shown in **Supplementary Table 2.**

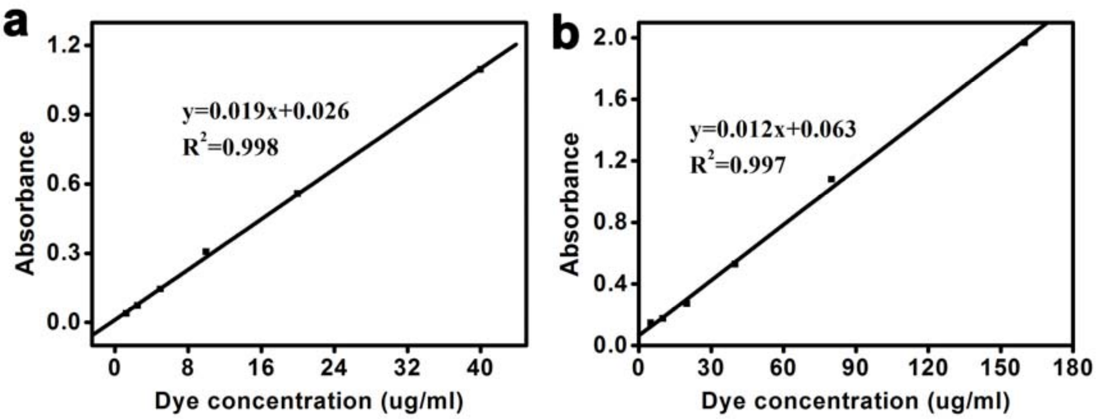

Supplementary Figure 9. The standard curve of ultraviolet absorption of dyes. (**a**) Nickel(II) phthalocyanine-tetrasulfonic acid tetrasodium salt; (**b**) Rhodamine B isothiocyanate.

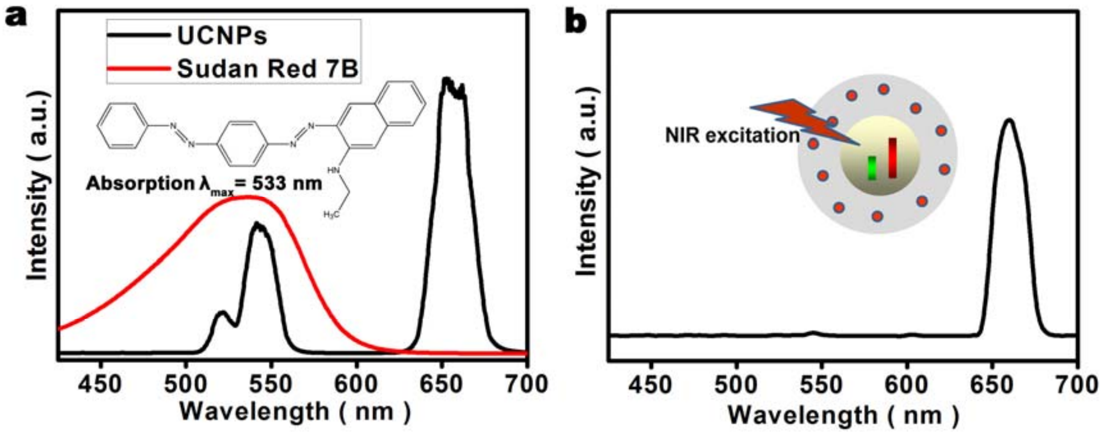

Supplementary Figure 10. Sb-UCNPs with non-fluorescent dyes-doped nanofitlers. Upconversion photoluminescence spectra of (**a**) *α*-$NaYbF_4$: 10 % Er@$NaYF_4$ nanoparticles. (**b**) After coating the sudan red 7B doped silica nanofilters on the *α*-$NaYbF_4$: 10 % Er@$NaYF_4$ nanoparticles, the green emission band can be filtered efficiently to realize the red emission sb-UCNPs.

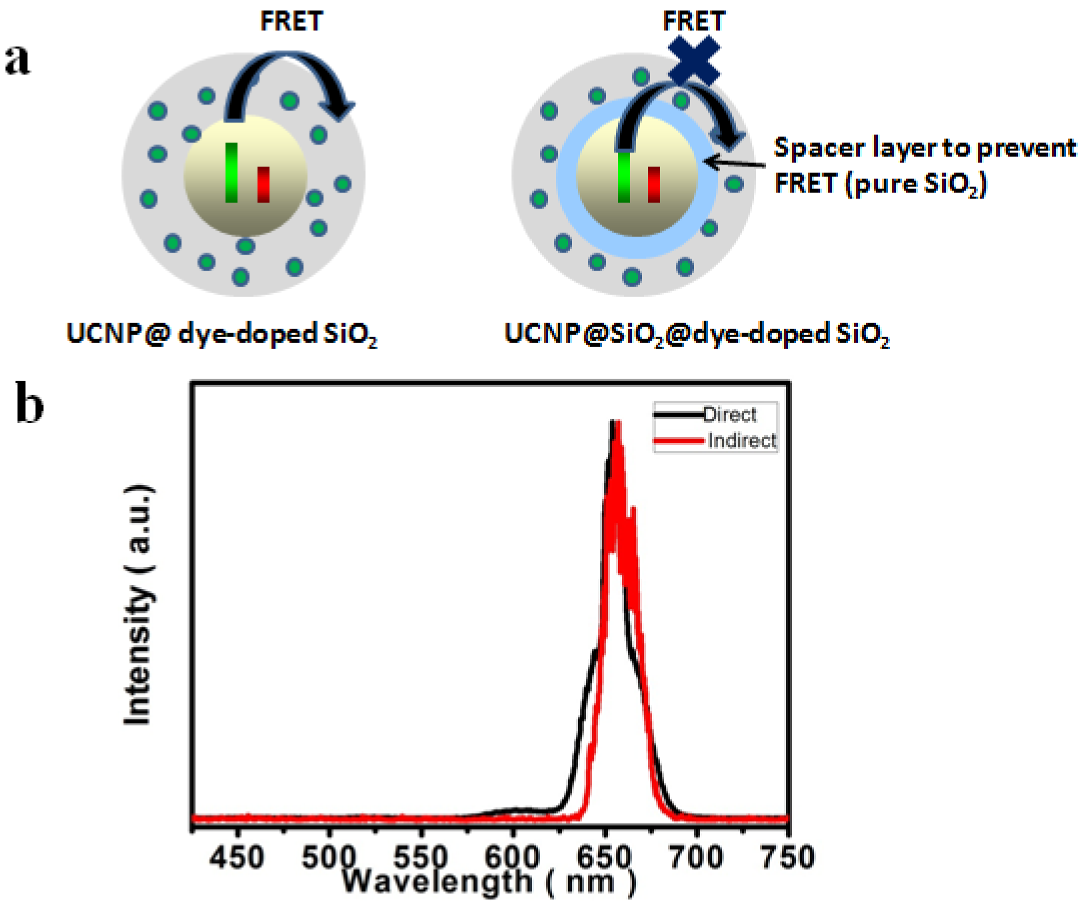

Supplementary Figure 11. Sb-UCNPs with fluorescent dyes-doped nanofitlers. (a) Scheme to show the way to avoid FRET between the upconversion emissions and fluorescent dyes by coating SiO_2 spacer layer. (**b**) UC spectra of *α*-$NaYbF_4$: 10 % Er@$NaYF_4$@rhodamine B isothiocyanate-doped SiO_2 nanoparticles (black) and *α*-$NaYbF_4$: 10 % Er@$NaYF_4$@SiO_2@rhodamine B isothiocyanate-doped SiO_2 nanoparticles (red).

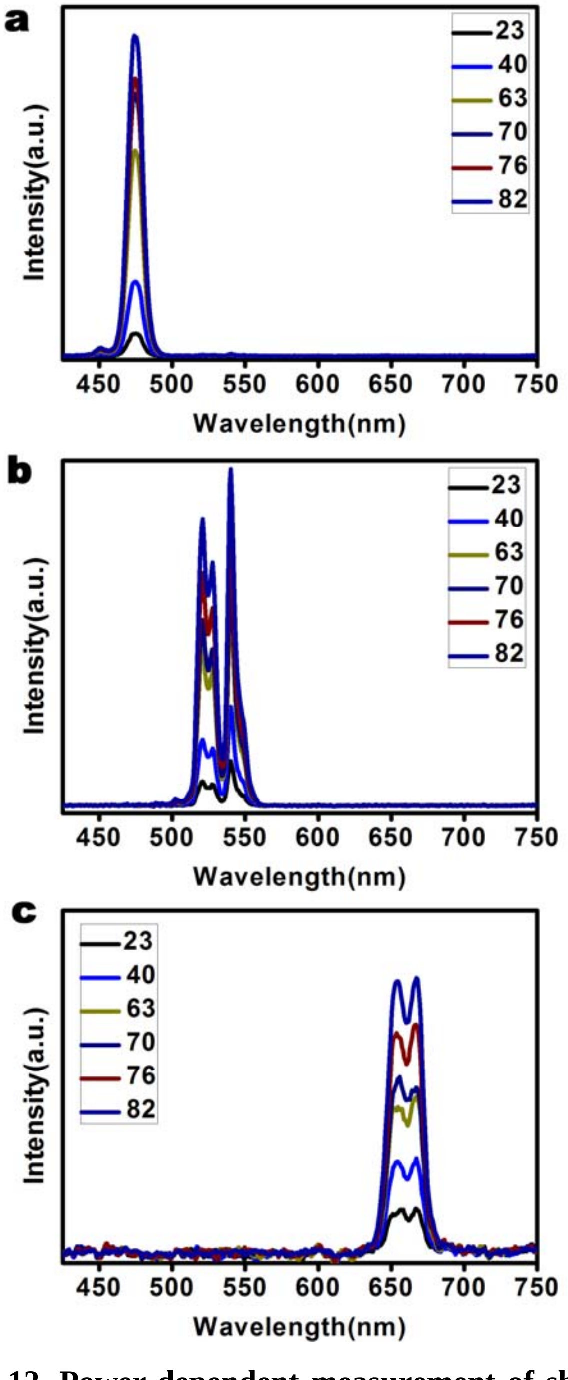

Supplementary Figure 12. Power-dependent measurement of sb-UCNPs. Upconversion photoluminescence spectra of the sb-UCNPs as a function of different power densities excitation with 980 nm laser (W/cm^2). (**a**) Blue, (**b**) green and (**c**) red sb-UCNPs. The single-band feature of the sb-UCNPs can be maintained even at the saturation point of upconversion emission intensity under the excitation with high power density (82 W/cm^2).

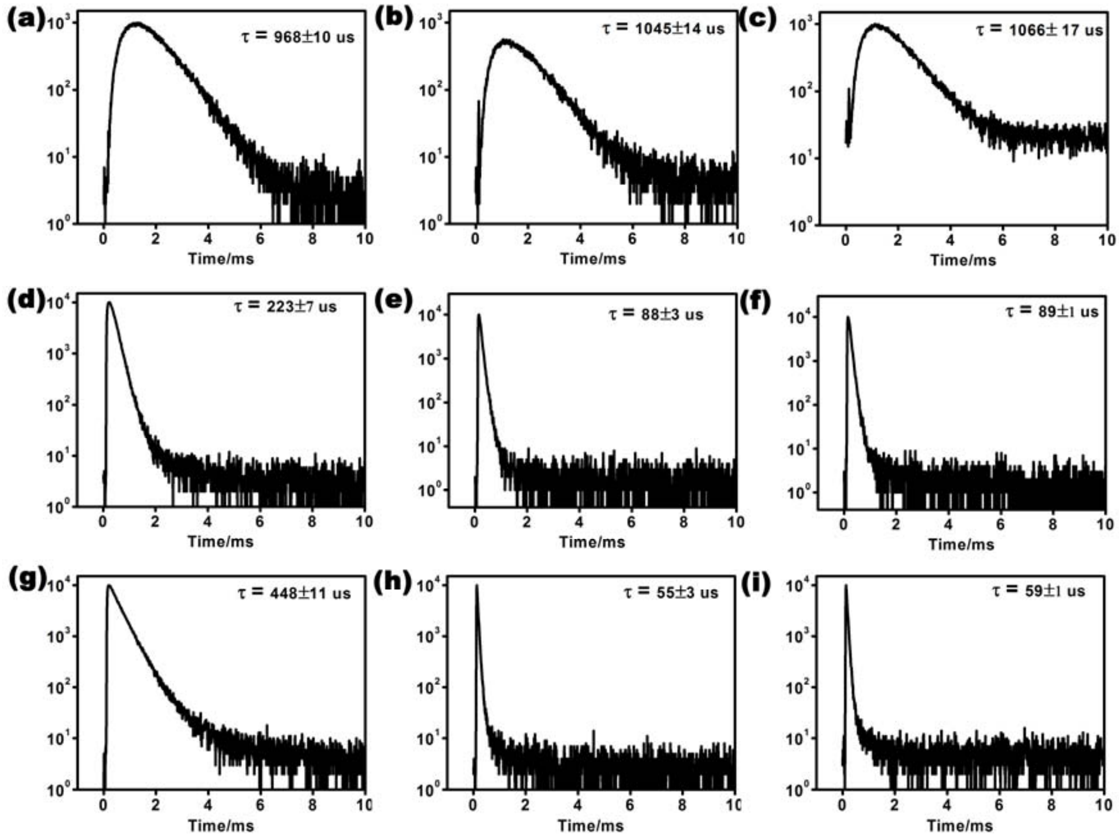

Supplementary Figure 13. Upconversion luminescence decay curves. The decay curves of UCNPs, UCNPs@SiO_2 and UCNPs@SiO_2@dye-doped SiO_2. (**a**) *β*-$NaGdF_4$: 20 % Yb, 0.2 % Tm@$NaGdF_4$ nanocrystals, (**b**) *β*-$NaGdF_4$: 20 % Yb, 0.2 % Tm@$NaGdF_4$@SiO_2 nanoparticles and (**c**) *β*-$NaGdF_4$: 20 % Yb, 0.2 % Tm@$NaGdF_4$@SiO_2@NPTAT-doped SiO_2. (**d**) *β*-$NaGdF_4$: 20 % Yb, 2 % Er nanocrystals, (**e**) *β*-$NaGdF_4$: 20 % Yb, 2 % Er@$NaGdF_4$@SiO_2 nanoparticles (**f**) *β*-$NaGdF_4$: 20 % Yb, 2 % Er@$NaGdF_4$@SiO_2@NPTAT-doped-SiO_2. (**g**) *α*-$NaYbF_4$: 10 % Er@$NaYF_4$ nanocrystals, (**h**) *α*-$NaYbF_4$: 10 % Er@$NaYF_4$@SiO_2 nanoparticles (**i**) *α*-$NaYbF_4$: 10 % Er@$NaYF_4$ @SiO_2@rhodamine B isothiocyanate-doped-SiO_2.

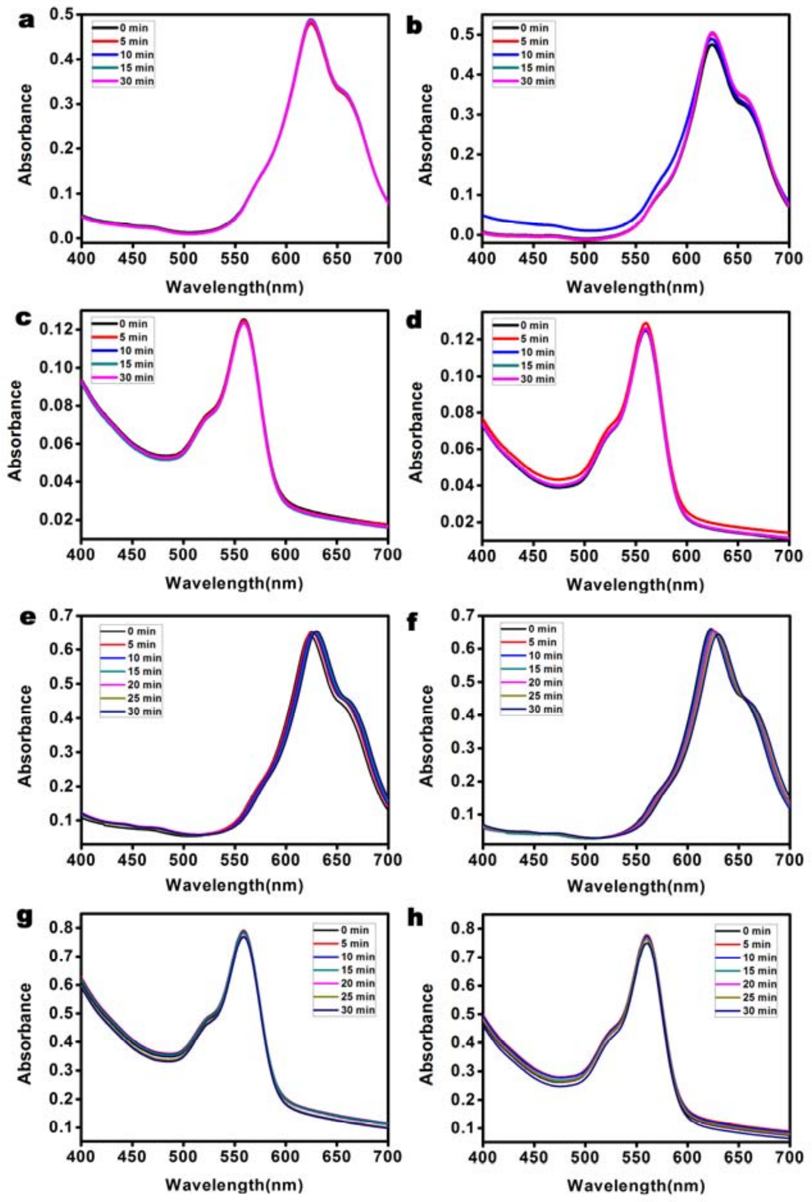

Supplementary Figure 14. The photostability of dyes doped in the nanofilters. UV-visible absorption spectrum of (**a**) β-$NaGdF_4$@NPTAT-doped SiO_2, (**b**) β-$NaGdF_4$: 20 %Yb, 0.2 %Tm@NPTAT-doped SiO_2, (**c**) α-$NaYbF_4$@rhodamine B isothiocyanate-dopedSiO_2, (**d**) α-$NaYbF_4$: 10 % Er@Rhodamine B isothiocyanate-dopedSiO_2 solution under continuous xenon lamp irradiation. UV-visible absorption spectrum of (**e**) β-$NaGdF_4$@NPTAT-doped SiO_2, (**f**) β-$NaGdF_4$: 20 %Yb, 0.2 %Tm@NPTAT-doped SiO_2, (**g**) α-$NaYbF_4$@rhodamine B isothiocyanate-–doped SiO_2, (**h**) α-$NaYbF_4$: 10 % Er@rhodamine B isothiocyanate-doped SiO_2 solution under continuous wavelength 980 nm laser irradiation.

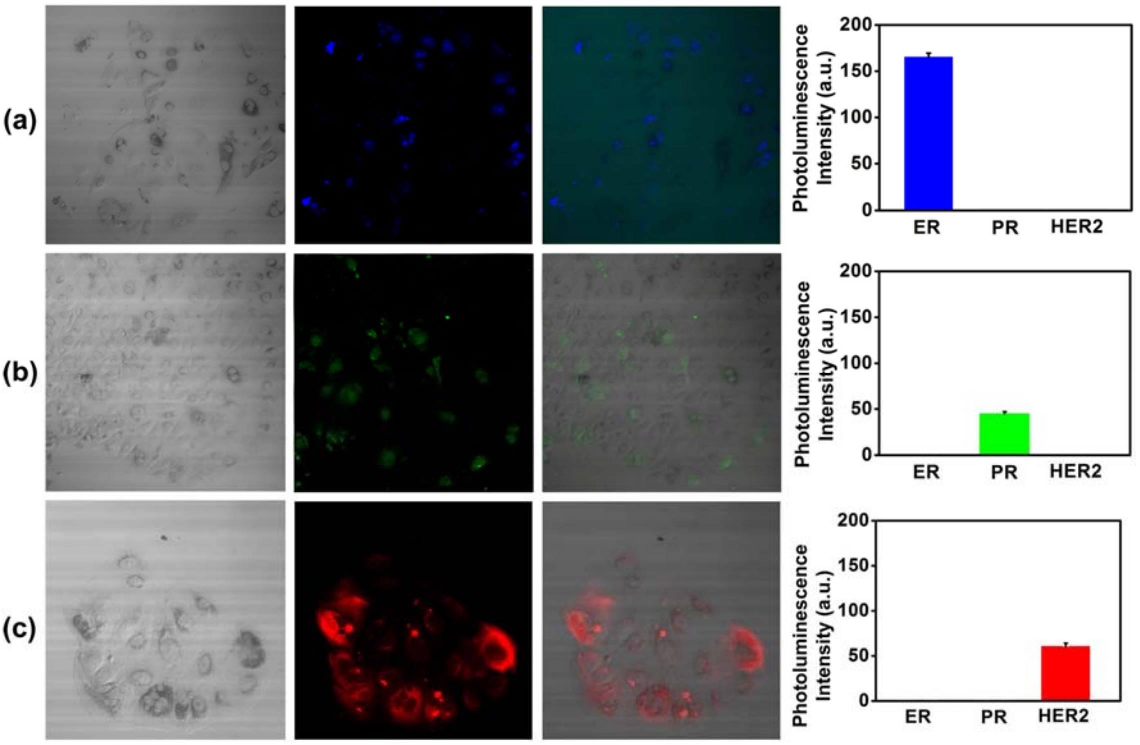

Supplementary Figure 15. Expression of ER, PR and HER2 biomarkers. Labeled by three kinds of sb-UCNPs respectively in MCF-7 breast cancer cells. (**a**) ER, (**b**) PR and (**c**) HER2. The signals were detected by fluorescence microscopy (pseudo-color). Average expression levels of (a, righ column) ER, (b, righ column) PR and (c, righ column) HER2 obtained from the results of spectral analysis of 100 single cells. Blue emission sb-UCNPs were used to labeled ER (a, left column), green emission sb-UCNPs were used to labeled PR (b, left column), and red emission sb-UCNPs were used to labeled HER2 (c, left column). In order to exclude the crosstalk effect between the three kinds of sb-UCNPs for the multiplexed detection, we also compared the photoluminescent intensities of individual color of each kind of sb-UCNPs labeled cells with that of multiplexed labeled cells. We demonstrated that there was no crosstalk between three kinds of sb-UCNPs when they were used for the multiplexed detection as shown in this figure.

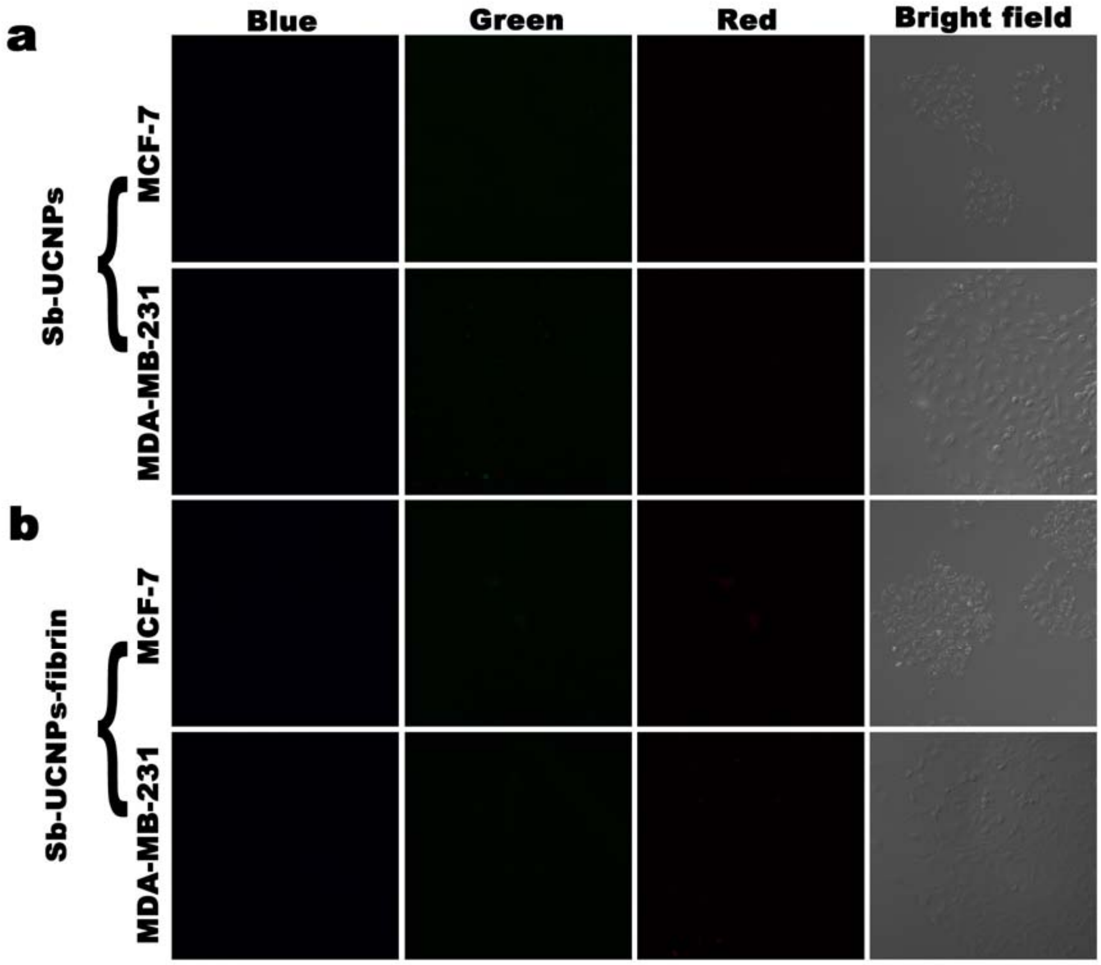

Supplementary Figure 16. Multiplexed detection of MCF-7 and MDA-MB-231 cell lines. (**a**) pure sb-UCNPs without antibodies modification and **(b**) sb-UCNPs conjugated with fibrinogen antibodies. Fibrinogen is rich in HepG2 cells but rare in breast cancer cells.

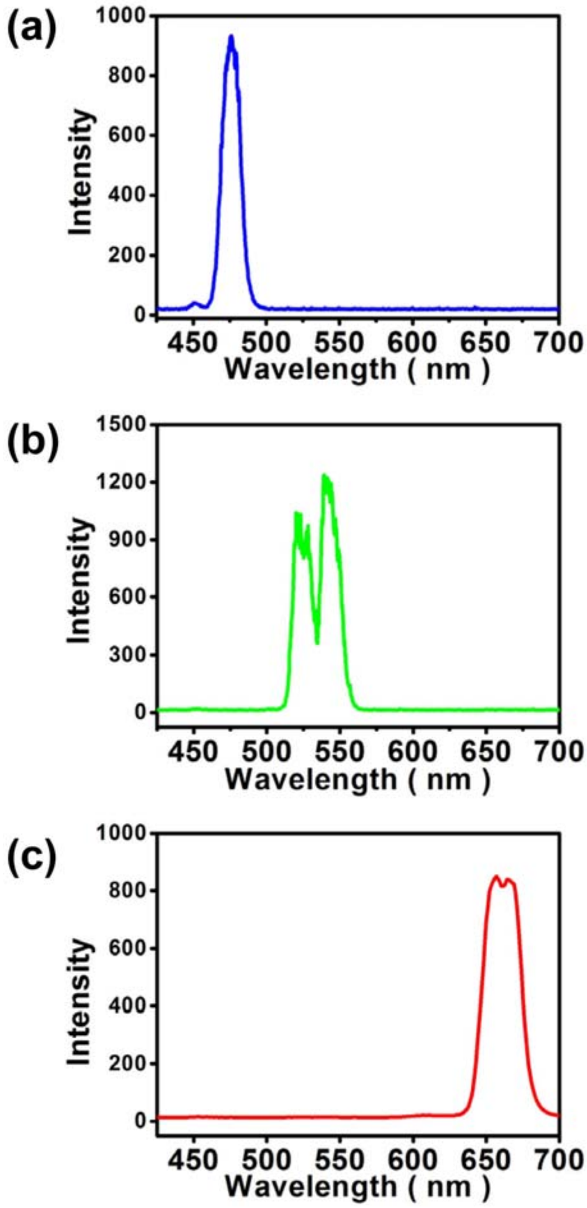

Supplementary Figure 17. The upconversion photoluminescence intensity of sb-UCNPs. Upconversion photoluminescence spectra of the blue (**a**), green (**b**) and red emission (**c**) sb-UCNPs with different emission intensity. (the three kinds of sb-UCNPs samples were dispersed in the solution with the same concentration to test the photoluminescence intensity under continuous wavelength 980 nm laser excitation with same power density (10 W/cm^2).

Supplementary Tables

Supplementary Table 1. Calculation of the doses of the precursors for the $NaGdF_4$:Yb,Er@$NaGdF_4$ UCNPs growth with different ML, and statistics average diameter and predict diameter of the obtained UCNPs with different ML growth.

Monolayers	Precursors (mL)		Average Measured Diameter (nm)	Predicted Diameter (nm)
	Re-OA (0.05 M)	Na-TFA-OA (2 M)		
0	-	-	2.50	-
1	0.10	0.05	3.32	3.22
2	0.15	0.08	3.89	3.94
3	0.21	0.11	4.55	4.66
4	0.30	0.15	5.41	5.38
5	0.38	0.19	6.22	6.10
6	0.48	0.24	6.91	6.82
7	0.60	0.30	7.58	7.54
8	0.72	0.36	8.42	8.26
9	0.85	0.425	9.11	8.98
10	1.00	0.50	9.89	9.70
11	1.20	0.60	10.53	10.42

Supplementary Table 2. Measurement of the doping amounts of the dyes for the silica coating with different feeding amounts.

**Feeding Amount (ug)**	**Dyes doping amounts in β-NaGdF$_4$, Yb 20 %, Tm 0.2 % @dye-doped SiO$_2$ (ug) and molar ratio of doped dyes to Tm**	**Dyes doping amounts in β-NaGdF$_4$, Yb 20 %, Er 2 % @ dye-doped SiO$_2$ (ug) and molar ratio of doped dyes to Er**	**Dyes doping amounts in α-NaYbF$_4$, Er 10 % @ dye-doped SiO$_2$ (ug) and molar ratio of doped dyes to Er**
**100**	**59 (3:10)**	**68 (7:200)**	**/**
**300**	**221 (11:10)**	**225 (11:100)**	**/**
**500**	**350 (9:5)**	**404 (1:5)**	**301 (11:100)**
**800**	**/**	**/**	**358 (67:1000)**
**1000**	**/**	**/**	**436(81:1000)**

Supplementary Table 3. Quantitative profiling of human breast cancer cells by sb-UCNPs and IHC methods.

	**ER**			**PR**			**HER2**		
	**Sb-UCNPs**		**IHC**	**Sb-UCNPs**		**IHC**	**Sb-UCNPs**		**IHC**
	**a.u.**	**%**	**%(**scale)	**a.u.**	**%**	**%(**scale)	**a.u.**	**%**	**%(**scale)
**MCF-7**	**167±6**	**100**	**90-100 (3+)**	**48±3**	**29**	**40-60 (2+)**	**64±6**	**38**	**40-60 (2+)**
**MDA-MB-231**	**18±2**	**11**	**0-20 (1+)**	**9±1**	**5**	**0-20 (1+)**	**7±1**	**4**	**0 (0)**

Supplementary Table 4. Quantitative profiling of human breast cancer tissue specimens by sb-UCNPs and IHC methods.

	**ER**			**PR**			**HER2**		
	**Sb-UCNPs**		**IHC**	**Sb-UCNPs**		**IHC**	**Sb-UCNPs**		**IHC**
	**a.u.**	**%**	**%(**scale)	**a.u.**	**%**	**%(**scale)	**a.u.**	**%**	**%(**scale)
**Biopsy 1**	**156±12**	**100**	**90-100(3+)**	**141±12**	**91**	**90-100(3+)**	**0**	**0**	**0(0)**
**Biopsy 2**	**107±9**	**72**	**60-80 (2+)**	**69±6**	**47**	**50-70(2+)**	**147±10**	**100**	**90-100(3+)**

Supplementary Methods

Materials

Gadolinium (III) chloride anhydrous ($GdCl_3$, 99.99 %), ytterbium (III) chloride anhydrous ($YbCl_3$, 99.9 %), yttrium(III) chloride anhydrous (YCl_3, 99.9 %), erbium (III) chloride anhydrous ($ErCl_3$, 99.9 %), thulium (III) chloride anhydrous ($TmCl_3$, 99.9 %), sodium trifluoroacetate (Na-TFA, 98 %), 1-octadecene (ODE, 90 %), oleic acid (OA, 90 %), Oleylamine (OAM， 70 %), Nickel(II) phthalocyanine-tetrasulfonic acid tetrasodium (NPTAT), rhodamine B isothiocyanate, sudan red 7B, CO-520 surfactant, tetraethyl orthosilicate (TEOS), (3-Aminopropyl) triethoxysilane (APTES), 3-(triethoxysilyl)propyl isocyanate (ICPTES) were purchased from Sigma-Aldrich. Sodium hydroxide (NaOH, 96 %), ammonium fluoride (NH_4F, 96 %), NH_3 H_2O (wt 30%), and trifluoroacetic acid (TFA, 99 %) were obtained from Beijing Chemical Reagents Co. Ltd. Yttrium(III) oxide (Y_2O_3, 99.99 %), ytterbium(III) oxide (Y_2O_3, 99.99 %), erbium(III) oxide (Er_2O_3, 99.99 %), and thulium(III) oxide (Tm_2O_3, 99.99 %) were obtained from Shanghai Yuelong Rare Earth New Materials Co., Ltd. Antibodies of HER2 (mouse monoclonal), Estrogen Receptor (ER (mouse monoclonal)), Progesterone Receptor (PR (mouse monoclonal)) were purchased from US Biological company. Antibodies of fibrinogen (goat polyclonal) were purchased from abcam company. All chemicals were used as received without any further purification.

Synthesis of β-$NaGdF_4$: 20 % Yb, 0.2 % Tm nanoparticles

The synthesis of β-$NaGdF_4$: 20 % Yb, 0.2 % Tm nanoparticles with a size of ~ 10 nm in this work was similar to that reported previously by van Veggel et *al.*[1] In a typical procedure, $GdCl_3$ (0.798 mmol), $YbCl_3$ (0.20 mmol), $TmCl_3$ (0.002 mmol), oleic acid (OA, 6.0 mL) and 1-octadecene (ODE, 15.0 mL) were mixed together and heated to 140 °C under vacuum until a clear solution formed, after that, the solution was cooled down to room temperature. A solution of NaOH (2.5 mmol) and NH_4F (4.0 mmol) in methanol (10 mL) was added and the mixture was stirred for half an h. The reaction mixture was then heated to 70 °C and maintained for half an h to remove the methanol. Afterward, the solution was heated to 285 °C and maintained for 100 min under a gentle argon flow. Subsequently, the solution was cooled down to room temperature and the nanoparticles were centrifuged and washed twice

with ethanol. The nanoparticles were finally dispersed in 10 mL of cyclohexane for further use.

Synthesis of *β*-$NaGdF_4$: 20 % Yb, 2 % Er nanoparticles

The synthesis of *β*-$NaGdF_4$: 20 % Yb, 2 % Er nanoparticles were carried out all the same as that of the *β*-$NaGdF_4$: 20 % Yb, 0.2 % Tm nanoparticles except 0.78 mmol of $GdCl_3$ were used instead of 0.798 mmol of $GdCl_3$ and 0.02mmol of $ErCl_3$ were used instead of 0.002 mmol of $TmCl_3$.

Synthesis of cubic phase *α*-$NaYbF_4$: 10 % Er nanoparticles

The trifluoroacetates (TFA) of Y, Yb, Tm, and Er were prepared by the procedure reported by Roberts et al.[2] The syntheses of the core nanoparticles in this work were similar to that reported previously by Chen et al.[3] In a typical procedure ($NaYbF_4$: 10 % Er, ~ 9 nm), 1.00 mmol of Na-TFA, 0.90 mmol of Yb-TFA, and 0.10 mmol of Er-TFA were dispersed in 16.0 mL of OA and 8.0 mL of OAM. The result solution was then heated at 120 °C under vacuum with magnetic stirring for 30 min to remove water and oxygen. Finally, the solution was heated to 290 °C at a rate of about 10 °C/min under Ar gas protection and kept at this temperature under vigorous stirring for about 30 min. Finally, the mixture was cooled to room temperature precipitated, centrifuged and washed twice with ethanol. The nanoparticles were finally dispersed in 10 mL of cyclohexane for further use.

Preparation of shell precursors

Y-OA (0.10 M) precursor: A mixture of YCl_3 (2.50 mmol), OA (10.0 mL), and ODE (15.0 mL) was loaded in a reaction container and heated at 140 °C under vacuum with magnetic stirring for 30 min to remove residual water and oxygen. Then, the colorless Y-OA precursor solution (0.10 M) was obtained.

Gd-OA(0.1 M) precursor: The synthesis of Gd-OA precursors were carried out all the same as that of the Y-OA precursor except 2.5 mmol of $GdCl_3$ were used instead of 2.5 mmol of YCl_3.

Na-TFA-OA precursor: A mixture of Na-TFA (4.00 mmol) and OA (10.0 mL) was loaded in a container at room temperature under vacuum with magnetic stirring to remove

residual water and oxygen. Then the colorless Na-TFA-OA precursor solution (0.40 M) was obtained.

Calculate the amount of shell precursor for the growth of each monolayer

The spherical concentric shell model (CSM) was employed to calculate the amount of shell precursor necessary for the growth of each monolayer (ML).[3] Because of the highly symmetric structure of $NaGdF_4$, the ML was inferred to a thickness equal to half the *c*-lattice parameter. Although the $NaGdF_4$ shell adopted here has also hexagonal structure ($P6_3/m$, a = 6.02 Å, c = 3.60 Å), the cation sites are of three types. A one-fold site occupied by RE^{3+}, another site occupied randomly by $1/2Na^+$ and $1/2RE^{3+}$, and a twofold site occupied randomly by Na^+ and vacancies.[42] Therefore, referral to a $NaGdF_4$, the ML could be taken to mean a thickness equal to the *c*-lattice parameter of the bulk material, 0.36 nm in the case of the hexagonal $NaGdF_4$. The required Gd-OA precursor amount for every layer of one

$$m_{layer(n)} = m_{particle(n)} - m_{particle(n-1)} = \rho(V_{particle(n)} - V_{particle(n-1)})$$
$$= \rho\frac{4}{3}\pi(r_{particle(n)} - r_{particle(n-1)}) \quad (1)$$

Density of the $NaGdF_4$ materials (ρ) is calculated as follows:

$$\rho = \frac{m}{V} = \frac{M}{(N_A * c * \frac{\sqrt{3}}{2}a^2)/N'} \quad (2)$$

Here M represents relative molecular mass of the material of $NaGdF_4$, N' means the number of $NaGdF_4$ units that one crystal cell contains and according to crystal structure of the hexagonal $NaGdF_4$, N' = 1.5. So, for $NaGdF_4$, the cell parameter is a = 6.02 Å, c = 3.60 Å, molecular weight (M) = 256.3, and ρ = 5.65 g/cm^3. For $NaYF_4$, cell parameter is a = 5.96 Å, c = 3.53 Å, molecular weight M = 187.9, and ρ = 4.31 g/cm^3.

Particle number of one molar (N) the $NaGdF_4$ (N) is calculated as follows:

$$N = \frac{V_{per\ molar}}{V_{per\ particle}} = \frac{\frac{M_0}{\rho_0}}{\frac{4}{3}\pi r_0^3} \quad (3)$$

Here, we supposed that the reactants were complete reacted after the reaction prolong 100 min (the synthesis of the core nanoparticles). So, in the above equation, r_0 = 5 nm. Because

the particle number is constant during the particle growth from smaller to bigger, so the particle number of the obtained initial seed, which was used for the synthesis of core/shell nanoparticles, is nearly the same as *N*. According to equation (1), (2), (3), the calculation of doses of the precursors for the core/shell nanoparticles growth with different ML are shown in Supplementary Table 1.

Synthesis of *β*-$NaGdF_4$: 20 % Yb, 0.2 % Tm@$NaGdF_4$, *β*-$NaGdF_4$: 20 % Yb, 2 % Er@$NaGdF_4$, *α*-$NaYbF_4$:10 %Er@$NaYF_4$ Core/Shell nanoparticles

The *β*-$NaGdF_4$: 20 % Yb, 0.2 % Tm@$NaGdF_4$ core/shell UCNPs were synthesized by using the successive layer-by-layer (SLBL) method with a minor modification.[3] In a typical process, 2.5 mL of the purified *β*-$NaGdF_4$: 20 % Yb, 0.2 % Tm, core cyclohexane solution (~10 nm, ~0.25 mmol) was mixed with 4.0 mL of OA and 6.0 mL of ODE. The flask was pumped down at 70 °C for 30 min to remove cyclohexane and any residual air. Subsequently, the system was switched to Ar flow, and the reaction mixture was further heated to 280 °C at a rate of ~20 °C/min. Then, 1 mL Gd-OA (0.2 M) and 0.5 mL Na-TFA-OA (0.8 M) host shell precursors were alternately introduced by dropwise addition at 280 °C. Injection of shell precursor cycles were performed 8 cycles. The ripening time between each injection was kept at 20 min. The synthesis of *β*-$NaGdF_4$: 20 % Yb, 2 % Er@$NaGdF_4$ nanoparticles were the same as the synthesis of *β*-$NaGdF_4$: 20 % Yb, 0.2 % Tm@$NaGdF_4$ core/shell nanoparticles. The synthesis of *α*-$NaYbF_4$: 10 % Er@$NaYF_4$ core/shell nanoparticles were the same as the synthesis of *β*-$NaGdF_4$: 20 % Yb, 0.2 % Tm@$NaGdF_4$ except Y-OA were introduced instead of Gd-OA.Finally, the obtained core/shell nanoparticles were precipitated and washed in the same way as the core nanoparticles and dispersed in cyclohexane for further use.

Synthesis of single-band upconversion nanoparticles (UCNP@SiO_2@dye-doped SiO_2) with the fluorescent dyes-doped nanofitlers

The UCNP@SiO_2@dye-doped SiO_2 were synthesized by a reversed-phase microemulsion method. In a typical synthesis of single-band red emission *α*-$NaYbF_4$: 10 %Er@rhodamine B isothiocyanate-doped SiO_2, a mixing solution of 0.5 mL surfactant CO-520, 16 mL cyclohexane and 1 mL 0.1mM *α*-$NaYbF_4$: 10 % Er@$NaYF_4$ solution in

cyclohexane was vigorously stirred for 15 min. After that, 2.1 mL CO-520, 0.2 mL NH_3 H_2O (30 wt%) were added into the solution, and the container was sealed and sonicated for 15 min until a transparent reversed-phase microemulsion was formed. Then, 0.1 mL TEOS was added into the solution. After vigorously stirring for 2 h and 0.1 mL APTES modified rhodamine B isothiocyanate solution (5 mg/mL) was added. After continuous stirring at room temperature at the speed of 750 rpm for two days, products were precipitated by adding ethanol. The collected precipitate was washed with ethanol twice and dispersed in ethanol for further use. The single-band blue (β-$NaGdF_4$: 20 %Yb, 0.2 %Tm@NPTAT–doped SiO_2) and green (β-$NaGdF_4$: 20 %Yb, 2 %Er@NPTAT-doped SiO_2) emission UCNPs were synthesized similar to the single-band red emission UCNPs using Nickel(II) phthalocyanine-tetrasulfonic acid tetrasodium (NPTAT) (5 mg/mL) as doping dyes.

Synthesis of single-band upconversion nanoparticles (UCNP@dye-doped SiO_2) with the non-fluorescent dyes-doped nanofitlers

With the synthesis of single-band red emission α-$NaYbF_4$: 10 % Er@sudan red 7B-doped SiO_2 as a typical example, the protocal is listed as following. Mixing solution of 0.5 mL surfactant CO-520, 15 mL cyclohexane and 1 mL 0.1mM α-$NaYbF_4$: 10 % Er@$NaYF_4$ solution in cyclohexane was vigorously stirred for 15 min. After that, 2.1 mL CO-520, 0.3 mL NH_3 H_2O (30 wt%) were added into the solution, and the container was sealed and sonicated for 15 min until a transparent reversed-phase microemulsion was formed. Then, 0.1 mL TEOS was added into the solution. After vigorously stirred for 30 min and 1 mL ICPTES modified Sudan red 7B solution (1 mg/mL) was added. (The Sudan red 7B (1 mg) were dissovled in cyclohexane with 30 uL ICPTES and heated at 60 °C for 30 min with magnetic stirring for modification.) After continuous stirring at room temperature at the speed of 750 rpm for 6 h, another 1 mL ICPTES modified Sudan red 7B solution (1 mg/mL) was added and aged for 2 days with continuous stirring. Products were precipitated by adding ethanol and washed with ethanol twice and dispersed in ethanol for further use.

Synthesis of amine-functionalized sb-UCNPs

A solution of sb-UCNPs (0.1 mmol) and APTES (0.02 mL, 0.085 mmol) in ethanol (10 mL) were stirred for 24h at room temperature. The products were isolated and purified by means of centrifugation, washed three times with ethanol and dried under reduced pressure.

Characterization of sb-UCNPs

Transmission electron microscopy (TEM) observations were performed on JEM-2100F transmission electron microscope with an accelerating voltage of 200 kV equipped with a post-column Gatan imaging filter (GIF-Tri-dium). The UC luminescence emission spectra were recorded on Edinburgh Fluorescence Spectrometer FLS980 instrument, but the excitation source using an external 980 nm semiconductor laser (Changchun New Industries Optoelectronics Tech. Co., Ltd.) with an optic fiber accessory, instead of the Xenon source in the spectrophotometer (Unless otherwise specified, all spectra were collected under identical experimental conditions). The UV/Vis spectra were recorded on Lambda 35 PerkinElmer.

sb-UCNPs-Abs Conjugation

The conjugation was realized through active ester maleimide-mediated amine and sulfhydryl coupling.[4] This procedure results in less nanoparticle aggregation in comparison with carbodiimide-mediated COOH-NH_2 condensation[5]. Antibodies targeting ER, PR and HER2 were conjugated to the single-band blue, green and red emission sb-UCNPs respectively. Briefly, BSA free antibodies (50 uL) at a concentration of ~0.2mg/mL were reduced with 20 mM of DTT to expose free sulfhydryls and purified by dialysis. At the same time, sb-UCNPs were activated using the hetero-bifunctional crosslinker, 4-(maleimidomethyl)-1-cyclohexancarboxylic acid N-hydroxysuccinimide ester (SMCC) for 18 h. The activated sb-UCNPs were mixed with the reduced Abs for 1 h. The bioconjugates were concentrated using ultrafiltration and purified by dialysis.

Laser-scanning confocal microscopy

Images of antibody conjugated sb-UCNPs were acquired using a confocal microscope (FV1000, Olympus) equipped with a 980 nm laser (SDL-980LM-500T, 500 mW, Shanghai Dream Lasers Technology). The CW laser emitting at was directed by the galvanometer

mirrors and then focused by an objective lens into the specimen. Light emitted from the location of the scanning spot was deflected by the galvanometer mirrors and then separated from the excitation by a reverse excitation dichroic mirror (Excitation DM, short-pass, edge at 850 nm, model 850DMSP, OMEGA), then passed through a confocal pinhole and a filter, and finally entered a photomultiplier tube (R6357 Enhanced model, HAMAMATSU, Japan) as a detector. To simultaneously visualize multicolor sb-UCNPs, the sb-UCNPs were excited with a laser line at 980 nm. To do this, samples containing single sb-UCNPs for every color were used to generate a lambda stack. This spectral library was stored and then used as references to digitally separate the spectral components for each pixel of the fluorescence micrograph.

Western blot

Cell extracts were prepared with RIPA kit (Bogoo). Load cell extracts and separate proteins on an SDS polyacrylamide gel. Transfer the electrophoresed proteins onto an 0.45um nitrocellulose filter (NC) membrane and incubate the membrane for 30 min at room temperature in 3 % BSA-TBST blocking solution. Then wash the membrane at room temperature with TBST for five times. Incubate the membrane overnight at 4 °C in blocking buffer containing primary antibody. Wash the membrane with TBST for five times. Incubate the membrane at room temperature for 40 min in blocking buffer containing an secondary reagent goat anti-mouse-IgG (H+L) HRP at 1:10000. Wash the membrane at room temperature for 6 times with TBST wash buffer. Detect with chemiluminescence reagents.

Immunohistochemistry staining for cells

Cells were grown on glass coverslips in 24-well plate for 24 h. Then cells were fixed using 4 % paraformaldehyde in PBS (pH 7.4) for 30 min at room temperature. Remove parafomaldehyde solution and rinse 3 times with PBS. Permeabilize cells with 0.3 Triton X-100 in PBS for 15 min. Remove Triton X-100 solution and rinse 3 times with PBS. Add 0.3 % H_2O_2-methanol solution and incubate for 10 min. Remove incubating solution and rinse 3 times with PBS. Block with 2 % BSA in PBS for 30 min at 37 °C to block non-specific hybridization. Add ER, PR, HER2 antibodies separately to cells on cover slips,

incubate 1 h at 37 °C. Remove incubating solution and rinse 3 times for 5 min with PBS. Incubate all slips with a dilution of HRP-labeled secondary antibody in PBS, 2 % BSA for 30 min at 37 °C. Remove incubating solution and rinse 4 times for 5 min with PBS. Immerse every cover slip in freshly prepared DAB solution. Couterstain cells in hematoxylin for 5 min, then rinse twice with tap water. Pick up cover slips with forceps and drain away excess buffer. Place cover slip cell layer down on a glass slide containing a drop of mounting medium. Then observe in microscopes.

Multiplexed immunohistochemical staining for tissue specimens with sb-UCNPs-Abs[5]

All the waxed specimens were subjected to a 4 um thick serial sectioning for immunohistochemical staining. After xylene dewaxing, the slides were de-benzened using con-ventional gradient ethanol and immersed in water. After incubation for 10 min with 3 % H_2O_2, the slides were washed thoroughly using PBS and placed in the phosphate-EDTA buffer solution (50 %, pH 9.0). Antigens were retrieved in the 121 °C high-pressure cooker for 5 min, followed by cooling down of the slides to room tempera-ture. Approximately, 1 mL of the ER, PR and HER-2 monoclonal antibodies conjugated sb-UCNPs (20 nM in PBS with 5 % albumin) were added to each slide. The slides were incubated with antibodies for 2 h at room temperature and washed with PBS. The slides were then washed 3 times with PBS. The slides were then washed with distilled water and sealed with neutral resin. The UCNPs staining slides were directly detected after incubation.

Immunohistochemical staining for tissue specimens[5]

ER, PR and HER2 were evaluated by IHC on formalin-fixed paraffin-embedded tissue. PR and ER were evaluated by the percentage of positive nuclei through visual microscopic estimation: <1 % negative, 1-9 % low positive, and ≥10 % positive. HER2 was evaluated by membrane staining through visual microscopic estimation and semi-quantitatively graded: 0, 1+ negative; 2+ equivocal; 3+ positive.

Supplementary References

1. Johnson, N. J. J., Oakden, W., Stanisz, G. J., Prosser, R. S. & van Veggel, F. C. J. M. Size-tunable, ultrasmall $NaGdF_4$ nanoparticles: insights into their T1 MRI contrast enhancement. *Chem. Mater.* **23**, 3714-3722 (2011).

2. Roberts, J. E. Lanthanum and neodymium salts of trifluoroacetic acid. *J. Am. Chem. Soc.* **83**, 1087-1088 (1961).

3. Li, X. M., Wang, R., Zhang, F. & Zhao, D. Y. Engineering homogeneous doping in single nanoparticle to enhance upconversion efficiency. *Nano. Lett.* **14**, 3634-3639 (2014).

4. Yezhelyev, M. V. *et al.* In situ molecular profiling of breast cancer biomarkers with multicolor quantum dots. *Adv. Mater.* **19**, 3146-3151 (2007).

5. Sun, L. *et al.* Expressions of ER, PR, HER-2, COX-2, and VEGF in primary and relapsed/metastatic breast cancers. *Cell Biochem. Biophys.* **68**, 511-516 (2014).
